# Supplementary material for: Regional Growth Rate Differences Specified by Apical Notch Activities Regulate Liverwort Thallus Shape
Source: Curr Biol. 2017 Jan 9;27(1):16–26. doi: 10.1016/j.cub.2016.10.056 (PMC5226888; doi:10.1016/j.cub.2016.10.056)
Supplement: Document S1. Supplemental Experimental Procedures, Figures S1–S5, and Tables S1–S3 [file mmc1.pdf]

Current Biology, Volume *27*

Supplemental Information

Regional Growth Rate Differences

Specified by Apical Notch Activities

Regulate Liverwort Thallus Shape

Jeremy E. Solly, Nik J. Cunniffe, and C. Jill Harrison

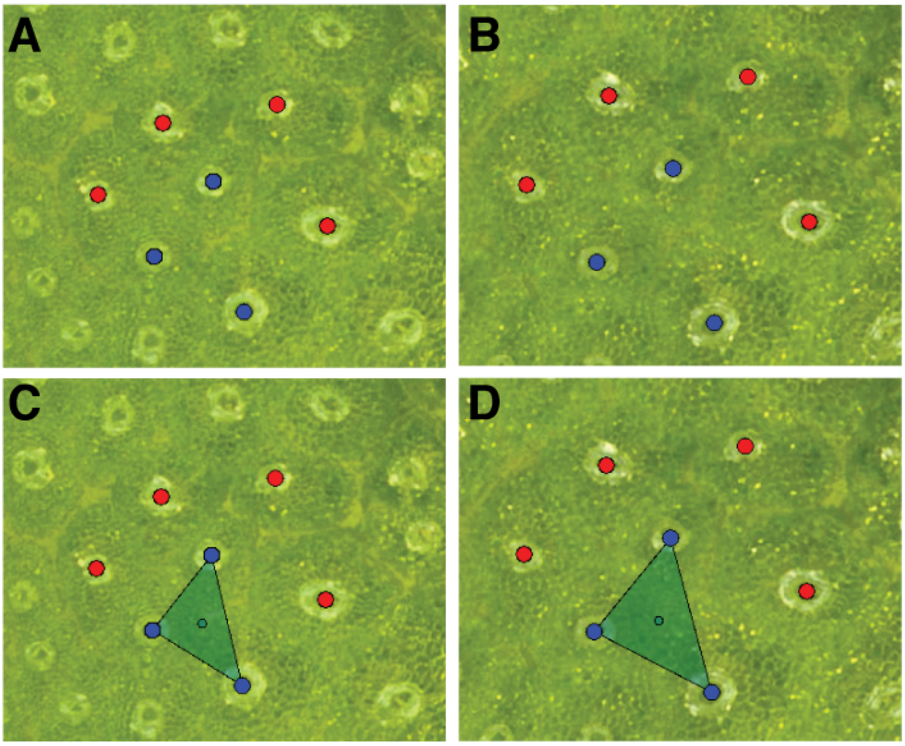

Figure S1 related to Figure 2: Quantification of thallus growth rates using Point Tracker software. (A,B) Points were manually placed on air pores in the first (A) and subsequent (B) images of a time series. (C, D) The points in blue demarcate a triangular region of thallus tissue, and the displacement of points over time (D) was used to calculate areal growth rates.

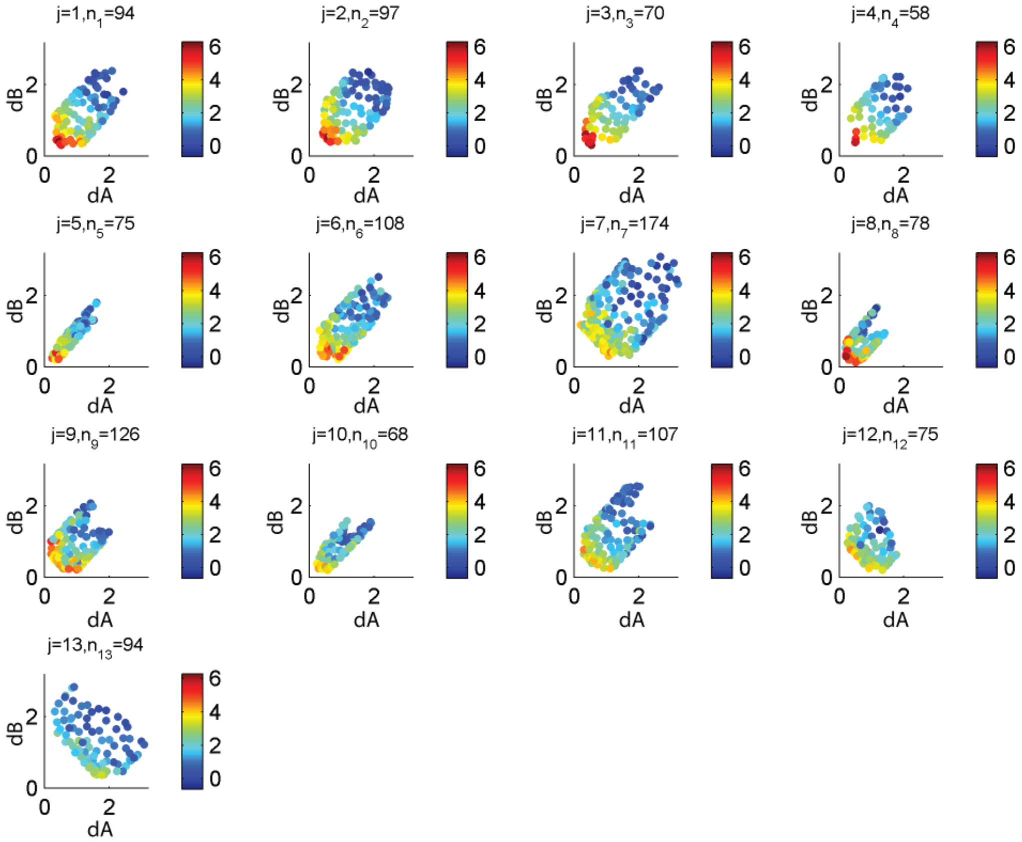

Figure S2 related to Figure 2: Growth rate distributions relative to apical notches generated from 13 half thalli. Each graph represents the growth rate data for one half-thallus over 24 h and dA and dB denote the distances from each notch in mm. j denotes dataset number and n_j denotes the number of points in each dataset. Percentage growth rates are represented by a colour scale with units per hour.

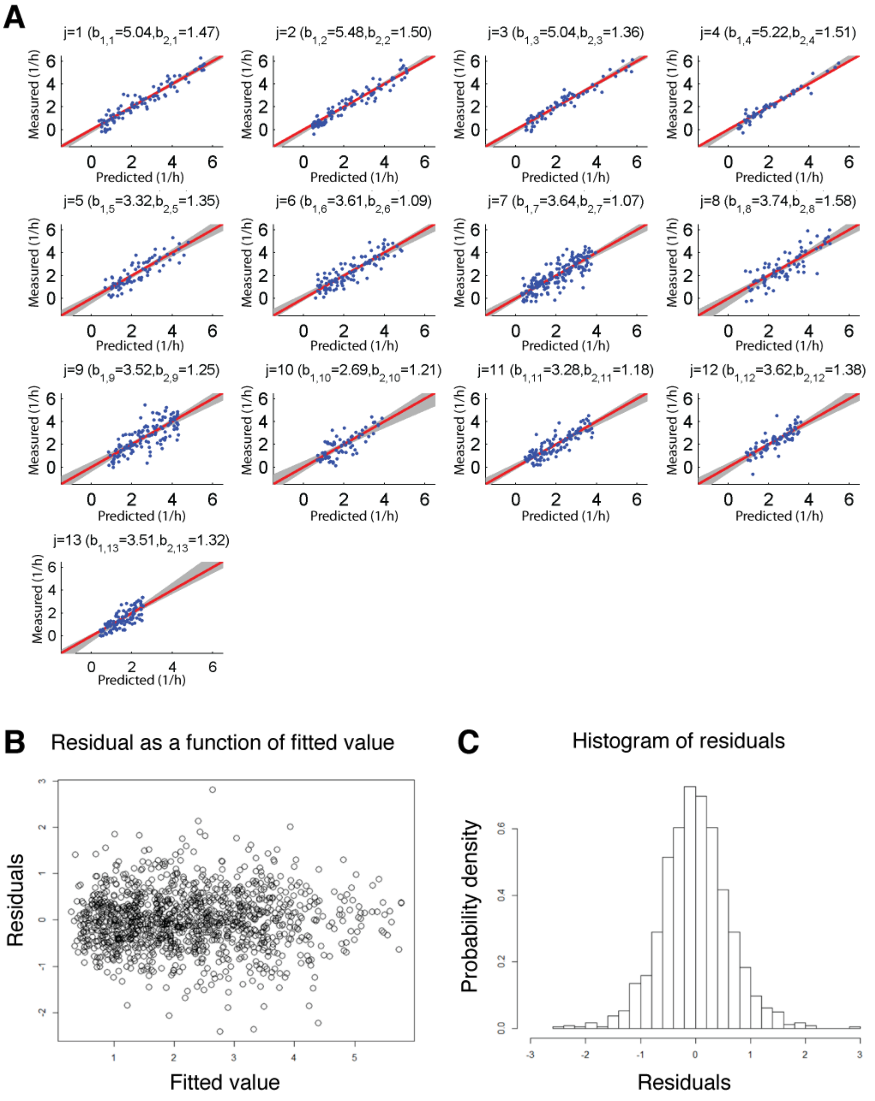

Figure S3 related to Figure 2: Fit of Model 2 (positional hypothesis, exponential decay) to all 13 datasets, showing the relationship between predicted and measured percentage growth rates and model diagnostics. (A) j denotes the dataset number. $b_{1,j}$ and $b_{2,j}$ denote the values of the parameters b_1 and b_2 used to fit the model to each dataset, j. The red lines show $y = x$, the values at which the measured and predicted growth rates exactly match. The grey shading shows the 95% confidence interval of the best-fit line for each dataset. As the red line always falls inside the grey region, the model predicted growth rates well across all 13 datasets. The general equation to calculate predicted growth rates was $k = b_1\left[\exp\left(-b_2 d_A\right) + \exp\left(-b_2 d_B\right)\right]$, where k denotes percentage growth rate, b_1 and b_2 denote parameters estimated to give the best fit for each dataset and d_A and d_B denote distance from notch A and notch B, respectively. (B) Residual-fitted plot, showing the size of residuals does not depend on the fitted value. (C) Empirical probability distribution for model residuals, showing the assumption of a normal distribution was reasonable, despite slight leptokurtic behaviour (a fat tail).

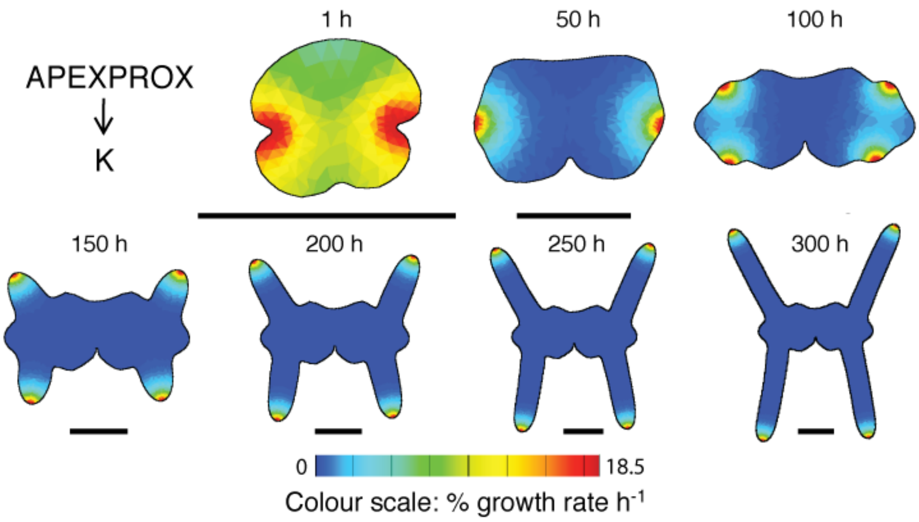

Figure S4 related to Figure 3: Growth suppression at APEX is required to maintain an invaginated notch in the notch-drives-growth model. A diffusible morphogen, APEXPROX was produced at APEX and distributed across the canvas. The model regulatory network is shown in the top left corner. The colour scale denotes percentage growth rate per hour. Scale bar =1 mm.

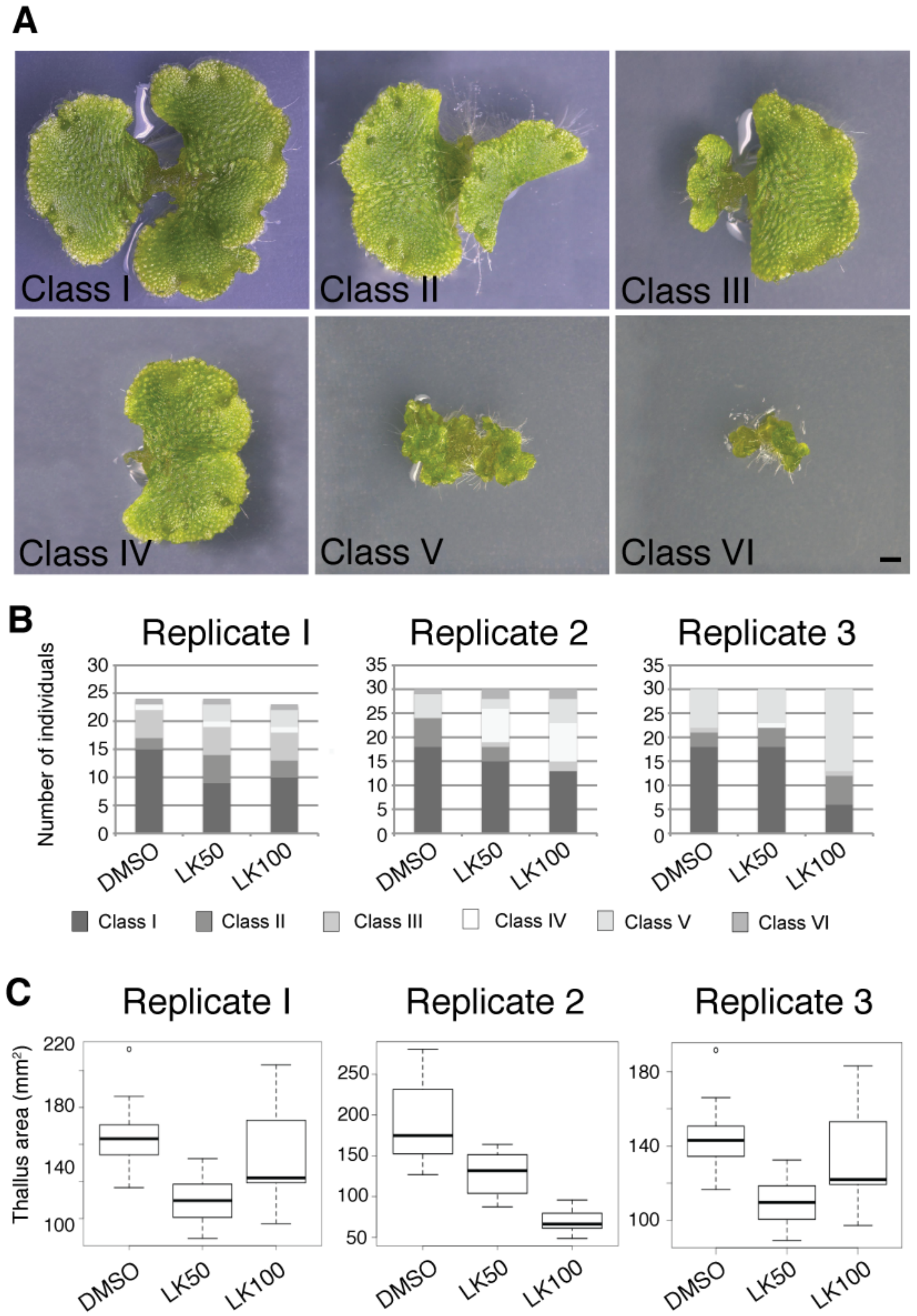

Figure S5 related to Figure 6: Developmental perturbations in thalli grown on L-kyn. (A) Class I thalli were normal, Class II thalli had polarity reversals, Class II thalli had a strong growth mismatch between thallus halves, Class IV plants only grew half a thallus, Class V thalli had differentiation defects and Class VI thalli didn't grow. Class I thalli from each treatment were included in growth analyses. Scale bar = 0.5 mm (B) The proportion of thalli with Class II to Class VI developmental defects varied between pharmacological treatments in three experimental replicates. (C) Class I thallus area varied by treatment in each experimental replicate.

	Model	Fitted parameters	ΔAIC	R^2
Model 1 (Mixed)	$k_{ij} = b_{1j} - b_{2j}\left(d_{A,ij} + d_{B,ij}\right) + \varepsilon_{ij}$<br>$\begin{pmatrix} b_{1j} \\ b_{2j} \end{pmatrix} \sim N\left(\begin{pmatrix} b_1 \\ b_2 \end{pmatrix}, \begin{pmatrix} \sigma_1^2 & \rho\sigma_1\sigma_2 \\ \rho\sigma_1\sigma_2 & \sigma_2^2 \end{pmatrix}\right)$<br>$\varepsilon_{ij} \sim N(0,\sigma^2)$	$b_1 = 5.00\,(4.59 - 5.42)$<br>$b_2 = 1.26\,(1.10 - 1.43)$<br>$\sigma_1 = 0.73\,(0.48 - 1.11)$<br>$\sigma_2 = 0.29\,(0.19 - 0.44)$<br>$\rho = 0.79\,(0.45 - 0.93)$	266.0	0.73
Model 1 (Pooled)	$k_i = b_1 - b_2\left(d_{A,i} + d_{B,i}\right) + \varepsilon_i$<br>$\varepsilon_i \sim N(0,\sigma^2)$	$b_1 = 4.58\,(4.47 - 4.70)$<br>$b_2 = 1.03\,(0.98 - 1.07)$	609.3	0.61
Model 2 (Mixed)	$k_{ij} = b_{1j}\left(\exp(-b_{2j}d_{A,ij}) + \exp(-b_{2j}d_{B,ij})\right) + \varepsilon_{ij}$<br>$\begin{pmatrix} b_{1j} \\ b_{2j} \end{pmatrix} \sim N\left(\begin{pmatrix} b_1 \\ b_2 \end{pmatrix}, \begin{pmatrix} \sigma_1^2 & \rho\sigma_1\sigma_2 \\ \rho\sigma_1\sigma_2 & \sigma_2^2 \end{pmatrix}\right)$<br>$\varepsilon_{ij} \sim N(0,\sigma^2)$	$b_1 = 3.98\,(3.46 - 4.49)$<br>$b_2 = 1.33\,(1.22 - 1.44)$<br>$\sigma_1 = 0.90\,(0.59 - 1.36)$<br>$\sigma_2 = 0.18\,(0.11 - 0.29)$<br>$\rho = 0.64\,(0.11 - 0.88)$	0	0.78
Model 2 (Pooled)	$k_i = b_1\left(\exp(-b_2 d_{A,i}) + \exp(-b_2 d_{B,i})\right) + \varepsilon_i$<br>$\varepsilon_i \sim N(0,\sigma^2)$	$b_1 = 3.54\,(3.39 - 3.69)$<br>$b_2 = 1.20\,(1.14 - 1.26)$	355.4	0.68
Model 3 (Mixed)	$k_{ij} = b_{1j} - b_{2j}d_{M,ij} + \varepsilon_{ij}$<br>$\begin{pmatrix} b_{1j} \\ b_{2j} \end{pmatrix} \sim N\left(\begin{pmatrix} b_1 \\ b_2 \end{pmatrix}, \begin{pmatrix} \sigma_1^2 & \rho\sigma_1\sigma_2 \\ \rho\sigma_1\sigma_2 & \sigma_2^2 \end{pmatrix}\right)$<br>$\varepsilon_{ij} \sim N(0,\sigma^2)$	$b_1 = 4.34\,(3.95 - 4.74)$<br>$b_2 = 2.53\,(2.15 - 2.90)$<br>$\sigma_1 = 0.70\,(0.46 - 1.05)$<br>$\sigma_2 = 0.66\,(0.43 - 1.01)$<br>$\rho = 0.81\,(0.49 - 0.93)$	433.8	0.69
Model 3 (Pooled)	$k_i = b_1 - b_2 d_{M,i} + \varepsilon_i$<br>$\varepsilon_i \sim N(0,\sigma^2)$	$b_1 = 4.06\,(3.96 - 4.16)$<br>$b_2 = 2.11\,(2.01 - 2.21)$	716.0	0.58
Model 4 (Mixed)	$k_{ij} = b_{1j}\exp(-b_{2j}d_{M,ij}) + \varepsilon_{ij}$<br>$\begin{pmatrix} b_{1j} \\ b_{2j} \end{pmatrix} \sim N\left(\begin{pmatrix} b_1 \\ b_2 \end{pmatrix}, \begin{pmatrix} \sigma_1^2 & \rho\sigma_1\sigma_2 \\ \rho\sigma_1\sigma_2 & \sigma_2^2 \end{pmatrix}\right)$<br>$\varepsilon_{ij} \sim N(0,\sigma^2)$	$b_1 = 6.28\,(5.35 - 7.21)$<br>$b_2 = 1.35\,(1.21 - 1.50)$<br>$\sigma_1 = 1.65\,(1.08 - 2.52)$<br>$\sigma_2 = 0.24\,(0.15 - 0.38)$<br>$\rho = 0.78\,(0.38 - 0.93)$	196.0	0.74
Model 4 (Pooled)	$k_i = b_1\exp(-b_2 d_{M,i}) + \varepsilon_i$<br>$\varepsilon_i \sim N(0,\sigma^2)$	$b_1 = 5.76\,(5.53 - 5.99)$<br>$b_2 = 1.26\,(1.20 - 1.33)$	492.9	0.65

Table S1 related to Figure 2. Comparison of models fitted in growth analyses. In the mixed models, k_{ij} is the fitted growth rate for the ith observation in the jth group (i.e. the ith location on the jth thallus), $d_{A,ij}$ is the distance from notch A for that observation, $d_{B,ij}$ is the distance from notch B, $d_{M,ij} = \min(d_{A,ij}, d_{B,ij})$, ε_{ij} is the error, and the parameters b_{1j} and b_{2j} vary with j via a distributional

assumption. In the pooled models the fitted growth rate for the ith observation (pooled over all datasets) is k_i, $d_{A,i}$ is the distance from notch A for that observation, $d_{B,i}$ is the distance from notch B, $d_{M,i} = \min(d_{A,i}, d_{B,i})$, the error is ε_i, and the parameters to be estimated are simply b_1 and b_2. Note that k_{ij} and k_i always have units 1/h, b_1 and b_{1j} always have units 1/h, and σ_1^2 always has units $(1/h)^2$. The units of other parameters depend on the model in question. In both variants of models 1 and 3, b_2 and b_{2j} have units 1/(h mm), and σ_2^2 has units $(1/(h\ mm))^2$. However, in both variants of models 2 and 4, b_2 and b_{2j} have units 1/mm, and σ_2^2 has units $(1/mm)^2$. All fitted values are reported with 95% confidence intervals in brackets. The value of R^2 reported for each fitted model was calculated as the squared correlation between the predicted and fitted values. ΔAIC is reported relative to the Model Two (mixed), the best-fitting model.

Parameter	Description	Value	Effect of changing value
$b_{apexprox}$	APEXPROX concentration at APEX	0.06	Same shape transitions, different timing, different canvas size (larger values give bigger canvas).
$D_{apexprox}$	APEXPROX diffusion rate	0.03 mm^2 h^{-1}	Same shape transtitions, different canvas size (larger values give bigger canvas).
$\mu_{apexprox}$	APEXPROX decay rate	0.8 h^{-1}	Same shape transitions, different canvas size (larger values give smaller canvas).
h_{apex}	Inhibition coefficient for APEX	5	The extent of notch invagination. The minimum value required to make a notch was used.
$t_{branching}$	Branching time	80 h	Affects the timing of shape transitions, with a minor effect on canvas shape.

Table S2 related to Figure 3: List of parameters used in the notch-drives-growth model, and effect of changing parameter values on thallus shape.

Parameter	Description	Value	Effect of changing value
$b_{apexprox}$	APEXPROX concentration at APEX	0.06	Same shape transitions, different timing, different canvas size (larger values give bigger canvas).
$D_{apexprox}$	APEXPROX diffusion rate	0.03 mm^2 h^{-1}	Same shape transtitions, different canvas size (larger values give bigger canvas).
$\mu_{apexprox}$	APEXPROX decay rate	0.8 h^{-1}	Same shape transitions, different canvas size (larger values give smaller canvas).
$\mu_{diftissue}$	DIFTISSUE decay rate	0.04 h^{-1}	Same shape transitions, different canvas size (larger values give smaller canvas).
h_{apex}	Inhibition coefficient for APEX	5	The extent of notch invagination. The minimum value required to make a notch was used.
$t_{branching}$	Branching time	80 h	Affects the timing of shape transitions, with a minor effect on canvas shape.

Table S3 related to Figure 5: List of parameters used in the notch-pre-patterns-growth model, and effect of changing parameter values on thallus shape.

Supplemental Experimental Procedures

Thallus shape modelling

Model overview

Liverwort thallus growth was modelled using the Growing Polarised Tissue (GPT) framework in *GFtbox*, a MATLAB application [S1]. Model runs involved simulating tissue growth by calculating the deformation of a canvas, which represented liverwort thallus tissue. The canvas was made up of a finite element mesh and the pattern of deformation depended on the distributions of growth-modulating factors over the canvas.

For both models described here, the initial canvas was formed in the shape of a gemma, consisted of around 440 elements and was roughly 0.5 mm wide. Simulations ran for up to 300 virtual hours and outputs were generated during growth. Finite elements were subdivided during growth.

Factors

Notional growth regulators were represented in the models as factors, which were distributed over the canvas as described elsewhere [S1]. Factors were either diffusible (able to propagate through the canvas) or fixed (immobile on the canvas). In equations, diffusible factors are denoted by a bold letter **s** subscripted with the factor name, while fixed factors are denoted by **i** subscripted with the factor name. For example, the diffusible factor APEXPROX is denoted by $\mathbf{s}_{apexprox}$ in equations, whereas the fixed factor APEX is denoted by $\mathbf{i}_{apex}$.

Initial distributions of factors were established during a setup phase from -9 h to 0 h. Factors had a single value for each vertex on the canvas and values between vertices were linearly interpolated across each finite element.

Parameters

The parameters and parameter values used in model simulations are shown in Tables S2 and S3. A range of parameter values for each parameter was explored, and values included here were selected by their capacity to reproduce shape transitions identified in Figure 1. The effect of varying parameter values was explored and is described in Tables S2 and S3.

Models

The pattern of deformation in each model was affected by the distributions of factors. Factors could interact with each other and influence specified growth rates. The model Gene Regulatory Network (GRN) determined the interactions between factors. The Growth rate Regulatory Network (KRN) determined how factors influenced specified growth rates.

GRN: 'notch-drives-growth' model

Two discrete regions of the fixed factor APEX were expressed at a level of one at the base of each apical notch on the initial canvas and zero elsewhere. The diffusible factor APEXPROX was fixed at a value of 0.06 ($b_{apexprox}$) where APEX was expressed and diffused according to the equation:

$$\frac{\partial \mathbf{s}_{apexprox}}{\partial t} = D_{apexprox} \nabla^2 \mathbf{s}_{apexprox} - \mu_{apexprox} \mathbf{s}_{apexprox} \text{ ,}$$

where $\mathbf{s}_{apexprox}$ is the concentration of APEXPROX, $D_{apexprox}$ is its diffusion rate and $\mu_{apexprox}$ its decay rate across the canvas. The APEXPROX distribution was allowed to establish during the setup phase for 9 h of virtual time before growth commenced and APEXPROX propagation continued throughout growth.

At time 80 h ($t_{branching}$), branching was imposed on the apices. The single region of APEX at the base of each notch was replaced by two discrete regions, which expressed APEX at a level of one. APEXPROX continued to be fixed at a concentration of $b_{apexprox}$ where APEX was expressed.

GRN: 'notch-pre-patterns-growth' model

The distributions of APEX and APEXPROX were identical in both models. Four new factors were included in the 'notch-pre-patterns-growth' model. TRANSITION was positioned in a ring around each region of APEX. It was expressed at a level of one in any vertex that was between 0.08 and 0.14 mm from the centre of APEX. TRANSITION defined whether canvas regions expressed the factors DIFFERENTIATED or UNDIFFERENTIATED. Regions that were closer to APEX than TRANSITION or included APEX expressed UNDIFFERENTIATED at a level of one and DIFFERENTIATED was zero. Regions that expressed TRANSITION or were further from APEX than TRANSITION expressed DIFFERENTIATED at a level of one and UNDIFFERENTIATED was zero.

TRANSITION also promoted the production of DIFTISSUE. In regions expressing TRANSITION, the concentration of DIFTISSUE was set as equal to the value of APEXPROX. DIFTISSUE subsequently decayed according to the equation:

$$\frac{\partial \mathbf{s}_{diftissue}}{\partial t} = -\mu_{diftissue} \mathbf{s}_{diftissue} \text{ ,}$$

where $\mathbf{s}_{diftissue}$ is the concentration of DIFTISSUE and $\mu_{diftissue}$ is its decay rate. The diffusion rate of DIFTISSUE was set as zero.

The initial distribution of DIFTISSUE was set up manually as an apical-basal gradient across DIFFERENTIATED-expressing tissue. Once the canvas deformation commenced at time 0 h,

DIFTISSUE was produced only in tissue expressing TRANSITION and decayed in tissue not expressing TRANSITION.

KRN: 'notch-drives-growth' model

In *GFtbox* growth is calculated using two equations, which specify growth rates parallel (K_{par}) and perpendicular (K_{per}) to a polarity gradient, if present. If no polarity is present, specified growth is isotropic and its value is the average of the outputs of the two growth equations. Polarity was not invoked in these models, so the two equations were identical. Here, K_{par} and K_{per} are generalised as K for simplicity.

In the 'notch-drives-growth' model, K was proportional to the concentration of APEXPROX across the canvas. The function *inh* was used to enable growth inhibition at APEX and took the form:

$$inh\left(h_{apex},\mathbf{i}_{apex}\right)=\frac{1}{1+h_{apex}\mathbf{i}_{apex}},$$

where h_{apex} is the inhibition of growth by APEX and took a value of 5. Thus, the growth equation was:

$$K=\mathbf{s}_{apexprox}\bullet inh\left(h_{apex},\mathbf{i}_{apex}\right).$$

KRN: 'notch-pre-patterns-growth' model

In the 'notch-pre-patterns-growth' model, growth was regulated differently depending on whether tissue expressed DIFFERENTIATED or UNDIFFERENTIATED. In UNDIFFERENTIATED tissue close to APEX, growth rate was proportional to APEXPROX. In DIFFERENTIATED tissue, growth rate was instead proportional to DIFTISSUE:

$$K=\left(\mathbf{s}_{apexprox}\mathbf{i}_{undifferentiated}+\mathbf{s}_{diftissue}\mathbf{i}_{differentiated}\right)\bullet inh\left(h_{apex},\mathbf{i}_{apex}\right).$$

Supplemental Reference

S1. Kennaway, R., Coen, E., Green, A., and Bangham, A. (2011). Generation of diverse biological forms through combinatorial interactions between tissue polarity and growth. PloS Computational Biology 7, e1002071.
