# Supplementary material for: Regional Growth Rate Differences Specified by Apical Notch Activities Regulate Liverwort Thallus Shape
Source: Curr Biol. 2017 Jan 9;27(1):16–26. doi: 10.1016/j.cub.2016.10.056 (PMC5226888; doi:10.1016/j.cub.2016.10.056)
Supplement: Document S2. Article plus Supplemental Information [file mmc3.pdf]

Article

Current Biology

Regional Growth Rate Differences Specified by Apical Notch Activities Regulate Liverwort Thallus Shape

Highlights

- *Marchantia* thalli undergo stereotypical shape transitions during development
- Growth rate heterogeneity is sufficient to account for thallus shape
- Growth suppression at the apices is required to maintain the notches
- Growth rate distributions are patterned with respect to the notches

Authors

Jeremy E. Solly, Nik J. Cunniffe, C. Jill Harrison

Correspondence

jill.harrison@bristol.ac.uk

In Brief

Tissue polarity and anisotropic growth are pivotal to the generation of planar growth forms in flowering plants and flies. Liverworts have an ancient planar growth form of mixed shoot and organ nature. Solly et al. show that growth rate heterogeneity patterned relative to the apical notches is sufficient to generate thallus shape.

CrossMark

Solly et al., 2017, Current Biology *27*, 16–26
January 9, 2017 © 2017 The Author(s). Published by Elsevier Ltd.
http://dx.doi.org/10.1016/j.cub.2016.10.056

CellPress

Current Biology
Article

Regional Growth Rate Differences Specified by Apical Notch Activities Regulate Liverwort Thallus Shape

Jeremy E. Solly,[1] Nik J. Cunniffe,[1] and C. Jill Harrison[1,2,3,*]
[1]University of Cambridge, Plant Sciences Department, Downing Street, Cambridge CB2 3EA, UK
[2]University of Bristol, School of Biological Sciences, 24 Tyndall Avenue, Bristol BS8 1TQ, UK
[3]Lead Contact
*Correspondence: jill.harrison@bristol.ac.uk
http://dx.doi.org/10.1016/j.cub.2016.10.056

SUMMARY

Plants have undergone 470 million years of evolution on land and different groups have distinct body shapes. Liverworts are the most ancient land plant lineage and have a flattened, creeping body (the thallus), which grows from apical cells in an invaginated "notch." The genetic mechanisms regulating liverwort shape are almost totally unknown, yet they provide a blueprint for the radiation of land plant forms. We have used a combination of live imaging, growth analyses, and computational modeling to determine what regulates liverwort thallus shape in *Marchantia polymorpha*. We find that the thallus undergoes a stereotypical sequence of shape transitions during the first 2 weeks of growth and that key aspects of global shape depend on regional growth rate differences generated by the coordinated activities of the apical notches. A "notch-drives-growth" model, in which a diffusible morphogen produced at each notch promotes specified isotropic growth, can reproduce the growth rate distributions that generate thallus shape given growth suppression at the apex. However, in surgical experiments, tissue growth persists following notch excision, showing that this model is insufficient to explain thallus growth. In an alternative "notch-pre-patterns-growth" model, a persistently acting growth regulator whose distribution is pre-patterned by the notches can account for the discrepancies between growth dynamics in the notch-drives-growth model and real plants following excision. Our work shows that growth rate heterogeneity is the primary shape determinant in *Marchantia polymorpha* and suggests that the thallus is likely to have zones with specialized functions.

INTRODUCTION

All plants share similar developmental constraints. Plant cells are bounded by a rigid cell wall, meaning that growth can only occur through cell division and expansion without cell movement. They also share a similar complement of gene families and have tissues that must carry out similar functions; for instance, nearly all plants exhibit planar growth to facilitate light capture for photosynthesis [1–3]. Despite these constraints, plants have evolved a wide variety of forms. Most knowledge of plant development has been gained from angiosperms, in which radial sporophytic shoots iteratively generate determinate planar lateral organs, such as leaves [4]. In contrast, in more ancient groups such as liverworts, the gametophytic body is dominant and may comprise a flattened mat of tissue undergoing indeterminate planar growth [5]. The wide evolutionary distance between these non-homologous plant body types with a similar overall growth habit and function offers the opportunity to ask whether the developmental mechanisms that regulate plant form can differ between plant groups or are fundamental to all plants.

The four components of growth that contribute to the shape of tissues that are mechanically connected are growth rate, growth anisotropy, growth orientation, and tissue rotation [6]. Final tissue shape is determined by the combined effects of all of the different regional growth patterns across a structure throughout development. Many different growth patterns can generate an identical shape, so tissue growth dynamics must be experimentally measured to determine how shape arises [7]. Sufficiency of growth parameters to generate given shapes is non-intuitive to estimate and requires a modeling approach [8]. By combining live imaging, clonal analysis, genetics, and computational modeling, mechanisms underlying shape generation in petals and leaves have been identified [7, 9–11]. For instance, a model that can account for petal shape requires growth rate heterogeneity along the proximo-distal axis and a divergent tissue-level polarity field [7]. Such a model can be framed in terms of an explicit growth regulatory network with genetic and polarity components, thereby allowing discrimination between different growth hypotheses that could generate identical organ shapes [10]. Used in a range of organs and species, this approach has identified a key role for tissue polarity in orienting anisotropy to generate diverse planar organ types in both plants and animals [7, 9–12].

Thallose planar growth forms, which are not differentiated into stems and leaves, are among the most ancient within the land plants and are characteristic of modern liverworts, such as *Marchantia polymorpha* [13–18]. *Marchantia polymorpha* has recently been adopted as a model system for molecular

 © 2017 The Author(s). Published by Elsevier Ltd.
This is an open access article under the CC BY license (http://creativecommons.org/licenses/by/4.0/).

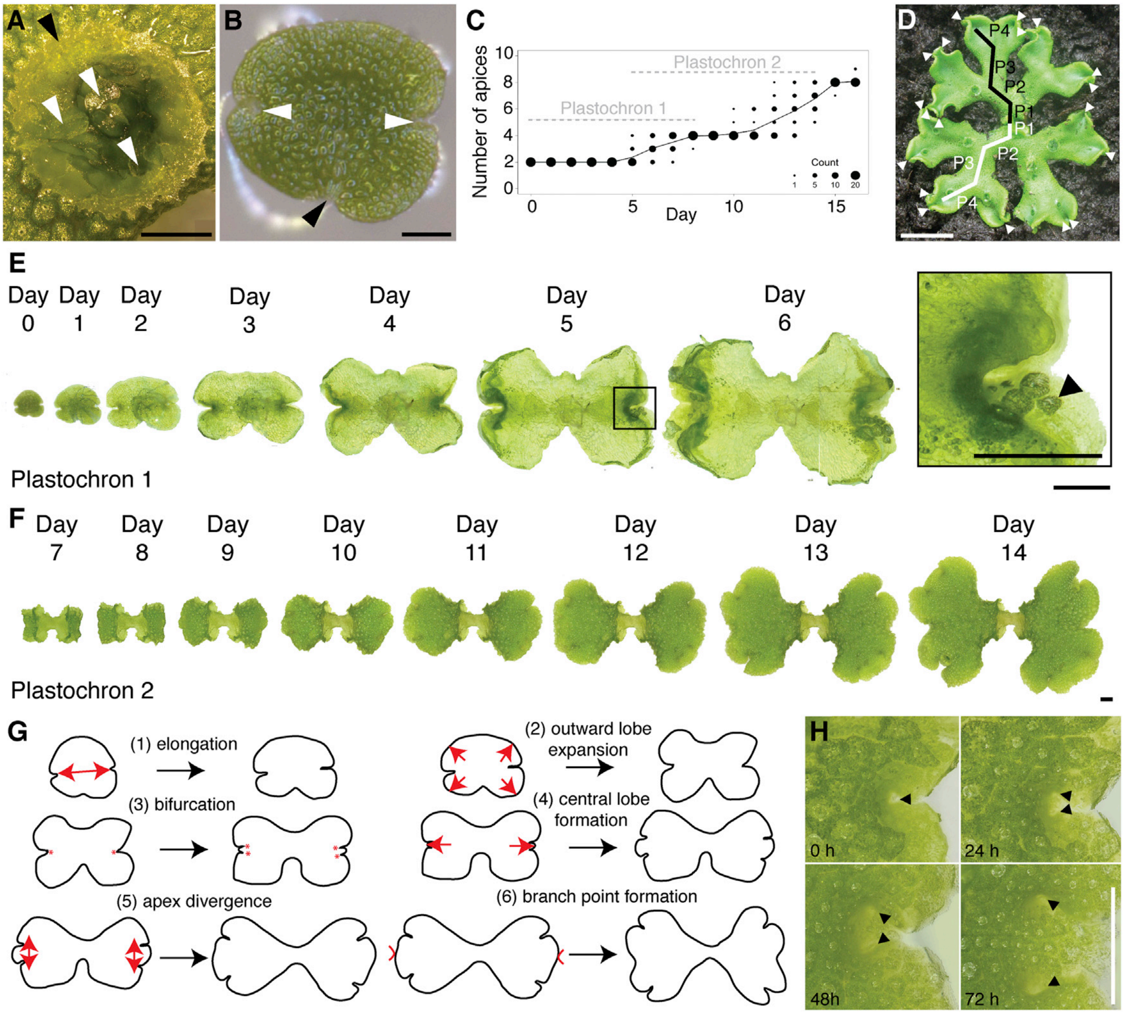

Figure 1. Shape Transformations in *Marchantia* Thallus Development

(A) A splash cup (black arrowhead) on the dorsal thallus showing gemmae with arrested development (white arrowheads). Scale bar, 1 mm.

(B) A gemma showing the stalk attachment point (black arrowhead) and notches at each end (white arrowheads). Scale bar, 100 μm.

(C) Bubble plot showing the change in notch number and plastochron over 16 days of growth.

(D) A 1-month-old plant showing the positions of notches (white arrowheads) and branches arising during plastochrons 1–4 (P1–P4). Scale bar, 1 cm.

(E) Time-lapse images of a plastochron 1 gemma grown over 6 days. The arrowhead in the inset shows the position of an air pore on the dorsal surface, marking mature tissue. Scale bar, 1 mm (inset, 0.5 mm).

(F) Time-lapse images of plastochron 2 taken on days 7–14 of growth. Scale bar, 1 mm.

(G) Schematics to illustrate key shape transitions occurring during plastochrons 1 and 2, as described in the main text.

(H) Time-lapse images showing bifurcation, central lobe formation, and notch divergence. Arrowheads show notches. Scale bar, 1 mm.

genetics, having the advantages of a small genome, low genetic redundancy, and susceptibility to *Agrobacterium*-mediated transformation [19, 20]. However, its development is poorly characterized and can be variable. Following a juvenile sporeling stage, new thallus tissue develops by the action of apical stem cell(s) located in invaginated notches at the thallus extremities, and these undergo periodic bifurcation to generate new axes of growth [5] (Figure 1). Here we have combined live imaging, growth analysis, and computational modeling to understand the growth dynamics that generate *Marchantia polymorpha* thallus shape. Our results suggest the differentiation of distinct functional zones oriented with respect to the apices, whose development may be under independent genetic control. Unlike studies of planar organ development in flowering plants or *Drosophila*, we have shown that growth rate heterogeneity is the primary shape determinant [7, 9–12].

RESULTS

Marchantia Grows as a Creeping Mat of Thallus Tissue that Bifurcates Regularly

To determine how the patterns of bifurcation and growth contribute to thallus morphogenesis, we undertook time-lapse imaging on plants grown from vegetative propagules called gemmae (Figures 1A and 1B). These initiate as cells at the tip of a short stalk in splash cups on the dorsal surface of mature

plants and comprise a disk of tissue with lateral, slightly indented apical notches (Figure 1B). Further gemma development is suppressed by the parent plant [21], and removal therefore allows a synchronous initiation of growth. We followed *Marchantia polymorpha* development by imaging gemmae each day for 16 days using a Keyence VHX-1000 series microscope. This identified bifurcation as a discrete and periodic event with characteristic timing, defined here as the notch plastochron (Figures 1C and 1D). The first plastochron covered the period between the onset of growth and the first bifurcation of each notch (6.6 days on average) to generate plants with a total of four notches (Figure 1C). During plastochron 1, a juvenile growth phase preceded a transition to adult growth, characterized by the appearance of thallus tissue with air pores on the dorsal surface (Figure 1E, inset). Plastochron 2 covered the period between the first and second bifurcations (6.9 days on average) to produce plants with a total of eight notches (Figures 1C, 1D, and 1F), and tissue generated in subsequent plastochrons resembled tissue generated in plastochron 2 (Figure 1D).

Key Thallus Shape Markers Arise during Plastochron 2

During plastochrons 1 and 2, the *Marchantia polymorpha* thallus undergoes a stereotypical sequence of shape transitions that determine overall plant form (Figures 1G and 1H). Gemma growth initiates by lateral expansion of tissue (Figure 1G; 1: the elongation phase), and the four lobes of the gemma then start to expand divergently (2; the lobe expansion phase). Bifurcation (3) then occurs to produce two apical notches on each side of the thallus, and these are separated by a small tongue of protruding tissue that we term the central lobe (4; central lobe formation). The central lobe subsequently expands, pushing the two notches apart (5; apex divergence). When the central lobe has ceased growth, it attains a concave rather than a convex shape with respect to adjacent notches, marking branch point formation (6). In plastochrons 3 and up, steps 3–6 are repeated in a similar pattern to the pattern shown in plastochron 2, allowing the thallus to spread radially (Figure 1D). As the shape transitions following the first bifurcation were typical of subsequent bifurcations, we reasoned that the patterns of growth during plastochrons 1 and 2 would represent the overall patterns of growth contributing to thallus shape. We therefore focused further growth analyses on these developmental stages.

Regional Growth Rate Differences Correlate with Notch Position

Whole thallus growth was quantified by measuring the area of plants grown from gemmae over a period of 2 weeks, and during the juvenile phase (plastochron 1) this was dominated by gemma expansion (Figures 2A and 2B). To quantify growth dynamics during plastochron 2, we used air pores on mature tissue (Figure 1E) as fixed-place tissue markers for image segmentation and growth analysis with Point Tracker software (Figure S1) [11]. This showed that areal growth rates were spatially heterogeneous across the thallus (Figure 2C). Rates were high in regions close to a notch, highest in regions between notches, and decreased to a negligible level at a distance of roughly 2 mm from a notch (Figure 2C). Thus, growth rates varied across the thallus depending on the proximity of tissue to the notch(es).

Tissue Growth Rates Depend on Position Relative to the Notches

The growth rate distributions above suggested long range action of the notches on tissue growth dynamics, and we identified two hypotheses that could potentially account for such action. One hypothesis (the positional hypothesis) was that tissue growth positively responds to a morphogen released by each notch. This positional hypothesis implies that tissue growth rates should depend jointly on the distance of tissue from both notches as both would contribute to the total concentration of morphogen received. A second hypothesis (the intrinsic hypothesis) was that tissue contains a counting mechanism to monitor when it was produced, such that more recently produced tissue grows faster. The intrinsic hypothesis implies that each notch acts autonomously to determine tissue growth rates. To discriminate between these hypotheses, we first plotted hourly growth rate during plastochron 2 against the distance from both notches (Figures 2D and 2E). Each dataset described the growth of half a thallus bearing two apical notches over a time period of roughly 24 hr and in total 13 datasets were analyzed (Figure S2). The nature of the relationship between growth rate and distance from the notches was not immediately clear, so statistical model fitting was undertaken to determine which hypothesis best explained the growth rate distribution data. The fit of four statistical models was evaluated, allowing for both exponential and linear responses of growth rate to distance:

model 1 (positional hypothesis) specified linear growth rate decay with distance from both notches, $k = b_1 - b_2(d_A + d_B)$;
model 2 (positional hypothesis) specified exponential growth rate decay with distance from both notches, $k = b_1[\exp(-b_2 d_A) + \exp(-b_2 d_B)]$;
model 3 (intrinsic hypothesis) specified linear growth rate decay with distance from the closest notch, $k = b_1 - b_2 d_M$; and
model 4 (intrinsic hypothesis) specified exponential growth rate decay with distance from the closest notch, $k = b_1 \exp(-b_2 d_M)$;

where k is the predicted growth rate, b_1 and b_2 are model coefficients, d_A and d_B are the distances from notches A and B, respectively, and $d_M = \min(d_A, d_B)$ is the distance from the closest notch. The coefficients b_1 and b_2 were fitted to experimental data, and they control the maximum growth rate and the rate at which the growth rate decreases with distance from the notches, respectively (see the Experimental Procedures). All models were symmetric in d_A and d_B as notch labeling was arbitrary.

In exploratory model fitting, a single model was fitted to the pooled data from all experimental datasets simultaneously. However, these analyses indicated that there was variability among the datasets, caused by systematic differences between thalli (Figure S2; Table S1). Therefore, non-linear mixed-effects regression was used in further fitting, allowing best-fitting model coefficients to vary between individual datasets. The fits of all models are summarized in Table S1.

The fits of the four mixed models were compared to each other using the Akaike Information Criterion (AIC) [22]. A lower AIC value indicates a better fit, and an AIC difference of ten or

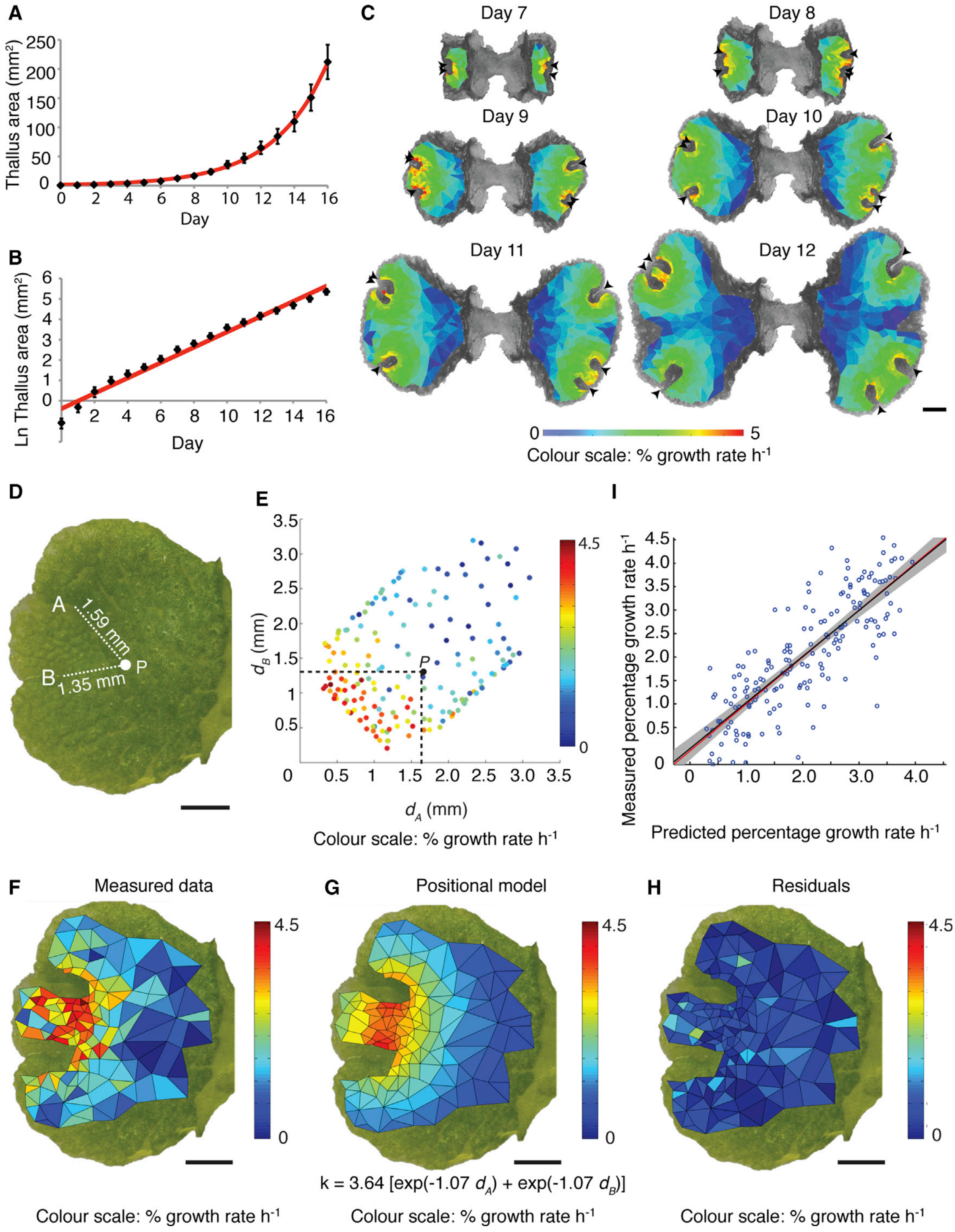

Figure 2. The Apical Notches Provide a Positional Cue that Regulates Tissue Growth

(A) Thallus area increases exponentially during the first 16 days of growth. The best-fit line follows the exponential curve $y = 1.5e^{0.31x}$ and $R^2 = 0.99$. Error bars represent the SD.

(B) Log-scaled version of the graph in (A) showing a slight decrease in growth rates over time. Error bars represent the SD.

(C) Heatmap of calculated areal growth rates plotted on meshes segmented from time-lapse images of a plant in plastochron 2. Black arrowheads indicate the apical notches. Color scale units are percentage growth rate per hour. Scale bar, 1 mm.

(D) Schematic to illustrate how distances from notches A and B were calculated for an example point (*P*) on a thallus. Scale bar, 1 mm.

(E) Scatterplot showing growth rates for each region of the thallus plotted against the distances from notches A and B.

(F) Measured regional growth rates plotted on a mesh segmented from an example thallus.

(legend continued on next page)

more between models suggests no support for models with higher values [23]. Model 2 was the best fit with the lowest AIC value by some margin ($AIC_1 - AIC_2 = 266.0$; $AIC_3 - AIC_2 = 433.8$; $AIC_4 - AIC_2 = 196.0$). Figures 2F–2H show the fit of model 2 to an example dataset (dataset 7). The model predictions corresponded well to the measured data (Figures 2F and 2G), as shown by the small residuals across the thallus and visual similarity between predicted and measured growth rates (Figures 2H and 2I). In addition to this example, model 2 predicted growth rates well across all 13 datasets with an R^2 value of 0.78 (Figures S2 and S3; Table S1). The experimental data therefore support the hypothesis that growth positively responds to a morphogen released by each notch, whose concentration decays exponentially with distance from the notch.

A Morphogen-Based Model of Thallus Development Can Reproduce Key Thallus Shape Transitions Only if Growth Is Inhibited at the Notch Itself

Although the growth analysis above suggests that the apical notches coordinate tissue growth dynamics remotely via the action of a diffusible cue, it samples only a subset of developmental stages and does not test sufficiency of a morphogen-driven growth hypothesis in generating the thallus shape changes identified in Figure 1. To test the hypothesis that a morphogen derived from each apex determines overall thallus shape, we used our results from live imaging in conjunction with computational modeling in the Growing Polarized Tissue (GPT) framework [8]. In this framework, growth-regulating factors are distributed over a canvas representing a sheet of tissue of given shape. Factors specify growth patterns across the canvas, and model simulations show the emergent shape changes that are produced by specified growth patterns (Figure 3).

A starting canvas was formed in the shape of a gemma, and apex position was specified with a fixed-place identity factor, APEX (Figure 3A). APEX produced a signaling factor, the diffusible morphogen APEXPROX, and APEXPROX underwent uniform decay across the canvas, resulting in a high concentration around the notch but lower concentrations elsewhere (Figure 3B). The designation of timing in model simulations was arbitrary, but parameters were selected such that 300 virtual hours resulted in model plants with similar shapes to real plants grown for 12 to 13 days. Bifurcation was included by splitting APEX into two at a specified time (80 virtual hours) and was therefore not an emergent property of models. We first specified a single growth rule: APEXPROX promotes isotropic growth. As the levels of APEXPROX tapered with distance from APEX, we hypothesized that this model should produce the growth patterns observed during live imaging. However, although the growth patterns around the apices were similar to those observed experimentally, this model was not able to capture any of the key shape transitions identified in Figure 1, but instead it gradually everted the apices from each notch (Figure S4). We therefore hypothesized that, as well as acting as a source of APEXPROX, APEX identity might suppress growth to maintain notch shape. The output of the model with these two growth rules was able to capture all of the shape transitions occurring during plastochrons 1 and 2 of thallus development (Figure 3C).

The "Notch-Drives-Growth" Model Is Insufficient to Explain Growth Dynamics after Surgical Apex Excision

If the notch-drives-growth model above is sufficient to account for plant shape, it should predict shape changes following experimental manipulations. One such manipulation that previously has been used to validate model predictions is surgical treatment [11]. We undertook surgical manipulations in the modeling framework by deleting regions of the canvas containing APEX at a time point in early plastochron 2 (150 virtual hours; Figure 4A). A prediction of the notch-drives-growth model is that, if the notches are the sole driver of growth, notch removal should rapidly lead to a cessation in growth (Figure 4A). APEXPROX-driven growth should persist for longest where the starting concentration is highest in the central lobe, and it should rapidly (by 155 virtual hours) diminish to zero by decay. Thus, a marked size differential should develop between the thallus halves over time (Figure 4A).

To undertake equivalent surgical experiments in plants, we grew plants from gemmae to plastochron 2 and imaged them. Plants were imaged again the next day, and the notches of half of each thallus were excised while leaving the other notches intact as a control (Figure 4B). Subsequent imaging was undertaken immediately after excision and then once a day over the next 3 days (Figure 4B). These surgical experiments showed that, as in the model a size differential developed between the two sides of the thalli over time, but the differential was smaller in plants than in the model (Figure 4B). The growth dynamics of tissue already present at the time of excision in each half thallus were quantified, and, as in the model, the highest growth rates were in the central lobe (Figure 4C). However, whereas modeled growth rapidly ceased, plant growth continued for at least 3 days with no apparent difference between intact and excised thallus halves (Figure 4C). A further difference in the outcomes of surgery between the model and real plants was that the cut edges of the thallus grew apart in plants but did not in the model. Therefore, a positional signal from the apical notch is insufficient to drive growth across the entire thallus.

A "Notch-Pre-patterns-Growth" Model Can Reproduce Key Shape Transitions and Thallus Responses to Surgical Apex Excision

The above results suggest that tissue growth rate depends on position relative to the apical notches, but they demonstrate that the notch-drives-growth hypothesis cannot fully account for growth. We reasoned that more persistent growth in the model could be generated by a differential response to

(G) The predicted pattern of regional growth rates is shown for the positional hypothesis with an exponential relationship between growth rate and distance. The best-fitting model equation is shown. k, growth rate; d_A and d_B, distance from notches A and B, respectively.

(H) Residuals (the differences between measured growth rates and those predicted by the best-fitting model) plotted for each region on the example thallus.

(I) The relationship between predicted and measured growth rates for the example thallus. The best-fit line is in black. The red line shows $y = x$, which falls within the 95% confidence interval of the best-fitting line, shown in gray.

See also Figures S1–S3 and Table S1.

CellPress

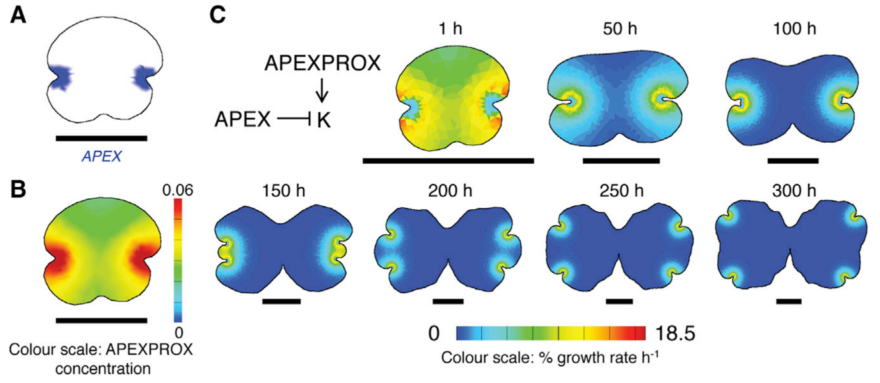

Figure 3. A Simple Model of Liverwort Growth Can Account for the Major Shape Transitions Occurring during Plastochrons 1 and 2

(A) The distribution of APEX on a gemma-shaped starting canvas. Scale bar, 0.5 mm.

(B) A diffusible morphogen, APEXPROX, was produced at APEX and diffused across the canvas. Color scale shows APEXPROX concentration in a.u. Scale bar, 0.5 mm.

(C) Growth regulatory network for the notch-drives-growth model and resultant thallus shapes at different time points. Color scale denotes percentage growth rate per hour. Scale bar, 1 mm.

See also Figure S4 and Table S2.

APEXPROX, whereby growth is set by APEXPROX in undifferentiated tissue but by other factors in differentiated tissue. This hypothesis was implemented in the model by adding new tissue identities and a new signaling factor. The identity factor TRANSITION was specified concentrically around APEX to mark a notional boundary between UNDIFFERENTIATED and DIFFERENTIATED tissue (Figures 5A and 5B). In UNDIFFERENTIATED tissue, the growth rate was set by the growth regulatory factor APEXPROX as previously described (Figure 5C). During growth, tissue was displaced from the apices through the TRANSITION domain. In TRANSITION, APEXPROX set the level of production of a second growth regulatory factor DIFTISSUE (Figure 5D), and DIFTISSUE then set the growth rate. DIFTISSUE did not diffuse but decayed slowly over time, and DIFFERENTIATED tissue growth rates therefore reflected the levels of DIFTISSUE remaining after decay. This model partially uncoupled growth set by APEXPROX from growth set by DIFTISSUE, and growth rates in the DIFFERENTIATED region diminished more slowly than in an equivalent region of the notch-drives-growth model.

The notch-pre-patterns-growth model had similar growth dynamics to the notch-drives-growth model and captured all of the shape transitions identified in Figure 1 (Figure 5E). Again we undertook surgical manipulations to validate the model, and we found that the notch-pre-patterns-growth model produced growth dynamics closely resembling those seen in real plants (Figure 5F). Growth persisted on the model canvas for 50–75 hr following notch excision in comparison to <5 hr in the notch-drives-growth model. The size differential between lobes was smaller than in the notch-drives-growth model and more closely resembled the differential in plants. As in real plants, the highest growth rates persisted in the central lobe and the cut site opened over time. The notch-pre-patterns-growth model was therefore sufficient to capture the major shape transitions occurring during normal thallus development, as well as responses to a surgical perturbation.

Reduced APEXPROX Concentrations and Auxin Synthesis Inhibition Affect Growth in Similar Ways

In both the notch-drives-growth and notch-pre-patterns-growth models, APEXPROX is the major determinant of growth, and reducing APEXPROX concentrations produces thalli that are small with low relative growth rates (Figure 6A). Homologs of the auxin biosynthetic genes *TRYPTOPHANAMINOTRANSFERASE OF ARABIDOPSIS* (*TAA*), *YUCCA*, and *STYLISH* are expressed in the apical notches in *Marchantia polymorpha*, and, among other defects, mutants have diminished plant size [24, 25]. Auxin is therefore a candidate generator of APEXPROX-like activity. To test the hypothesis that auxin can contribute to thallus growth in a way that is similar to APEXPROX, we treated plants with a pharmacological inhibitor of TAA-mediated auxin biosynthesis, L-kynurenine (L-kyn) (Figures 6B–6E; Figure S5). In three experimental replicates, thalli were grown for 14 days from gemmae on medium containing 0, 50, or 100 μM L-kyn (Figures 6B–6E; Figure S5). While many thalli had developmental defects (Figure S5) affecting polarity (class II defects), relative lobe growth (class III and IV defects), and differentiation (class V defects) or didn't grow (class VI defects), a subset of thalli grew normally (class I thalli) and showed a progressive, dose-dependent reduction in thallus size (Figures 6B–6D; Figure S5). Thus, as in modeling with reduced APEXPROX concentrations, L-kyn treatment inhibited overall thallus growth rates.

Qualitative comparison of model outputs showed that simulated thalli with lower concentrations of APEXPROX had lower maximum growth rates around the notches and smaller distances between the notches and thallus regions with negligible growth than thalli with higher APEXPROX concentrations (Figure 6A). To determine whether L-kyn-treated thalli showed similar qualitative changes, we selected thalli with similar sizes from each treatment class and quantified growth (Figure 6E). While a DMSO control had the highest growth rates around the notches, L-kyn-treated thalli had lower growth rates, and the distance between notches and thallus regions with negligible growth appeared smaller in L-kyn-treated plants. The data above show that pharmacological inhibition of TAA-mediated auxin production can have a similar effect on thallus growth to the predicted effect of APEXPROX depletion, and they support the hypothesis that auxin synthesis at the notch determines thallus shape.

DISCUSSION

We demonstrate that the *Marchantia polymorpha* thallus undergoes a stereotypical sequence of shape transitions during the first 2 weeks of growth and show that regional growth rates across the thallus correlate with the position of the apical notches. We have used computational modeling in combination with surgical and pharmacological experiments to gain insights into the mechanisms regulating thallus shape and have shown

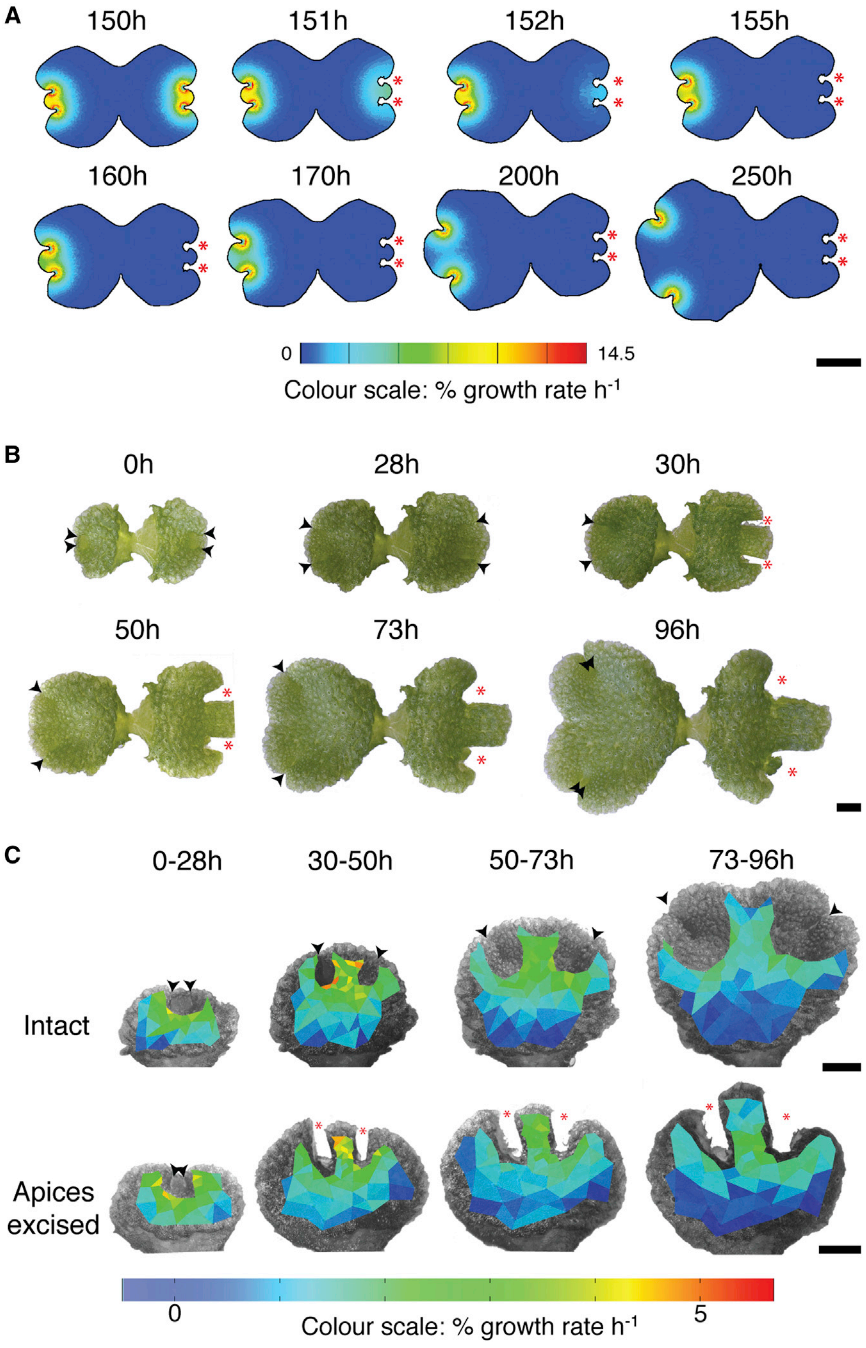

Figure 4. The Notch-Drives-Growth Model Is Insufficient to Account for Thallus Shape Changes following Surgery

(A) Predicted effects on thallus shape of notch excision (red asterisks) 150 hr into a model simulation. Growth drops off rapidly, resulting in a notable size differential between thallus halves. The color scale represents percentage growth rate per hour. Scale bar, 1 mm.

(B) Notch excision from half thalli at time 30 hr did not cause growth to cease, rather the cut site edges gradually diverged and the central lobe grew out. Black arrowheads indicate notches and red asterisks indicate cut sites. Scale bar, 1 mm.

(C) The growth rate distributions in tissue present at the time of excision were plotted, and they showed no conspicuous difference between uncut control thallus halves and halves with the apices excised. The color scale represents percentage growth rate per hour. Scale bar, 1 mm.

that a notch-drives-growth model can reproduce the major shape transitions occurring during thallus development given growth suppression at the apex. While this model is insufficient to capture growth responses to surgical apex excision, a notch-pre-patterns-growth model, in which the notches pattern the production of a persistent and autonomously acting growth regulator, is sufficient. This model is the first experimentally validated hypothesis for the molecular regulation of growth in a thalloid land plant and provides an explicit set of testable hypotheses for future work: (1) cellular growth rates at the apex should be low, (2) an apex-derived mobile signal regulates growth, and (3) growth of differentiated tissue is determinate. The morphogen APEXPROX is the primary shape determinant, and pharmacological inhibition of auxin biosynthesis can have similar effects to APEXPROX depletion in modeling. The data predict a morphogen-like action [26, 27] for auxin in thallus shape determination, with an auxin concentration gradient radiating from the notch and concentration-dependent cellular variation in growth rates emerging as an outcome of auxin action.

The genetic networks that determine *Marchantia polymorpha* thallus morphogenesis are not yet well known, so there is sparse molecular evidence in support of the above hypotheses. While expression of *MpTAA* and *MpYUC2* at the notch demonstrate that auxin production occurs in the right place for auxin to act as APEXPROX, mutants with defective *MpTAA* or *MpYUC2* activity have gross morphological perturbations comprising either an undifferentiated cell mass or a hyperbranched thallus [25]. However, selection for transformants with milder defects may reveal expression level-dependent size variation in future work. Auxin applied at low concentrations can stimulate thallus growth, but auxin application has pleiotropic effects, most notably profound growth inhibition and dorsoventral defects, such as ectopic rhizoid specification [28, 29]. Interference with auxin conjugation by constitutive overexpression of the bacterial *iaaL* gene causes severe stunting and loss of differentiated cell fates [24]. Narrower overexpression from a notch-specific promoter compromises thallus lobe growth and branching [24]. While the latter mutant phenotypes are consistent with a role for auxin at APEX or as APEXPROX, the free auxin levels in these lines have not been quantified.

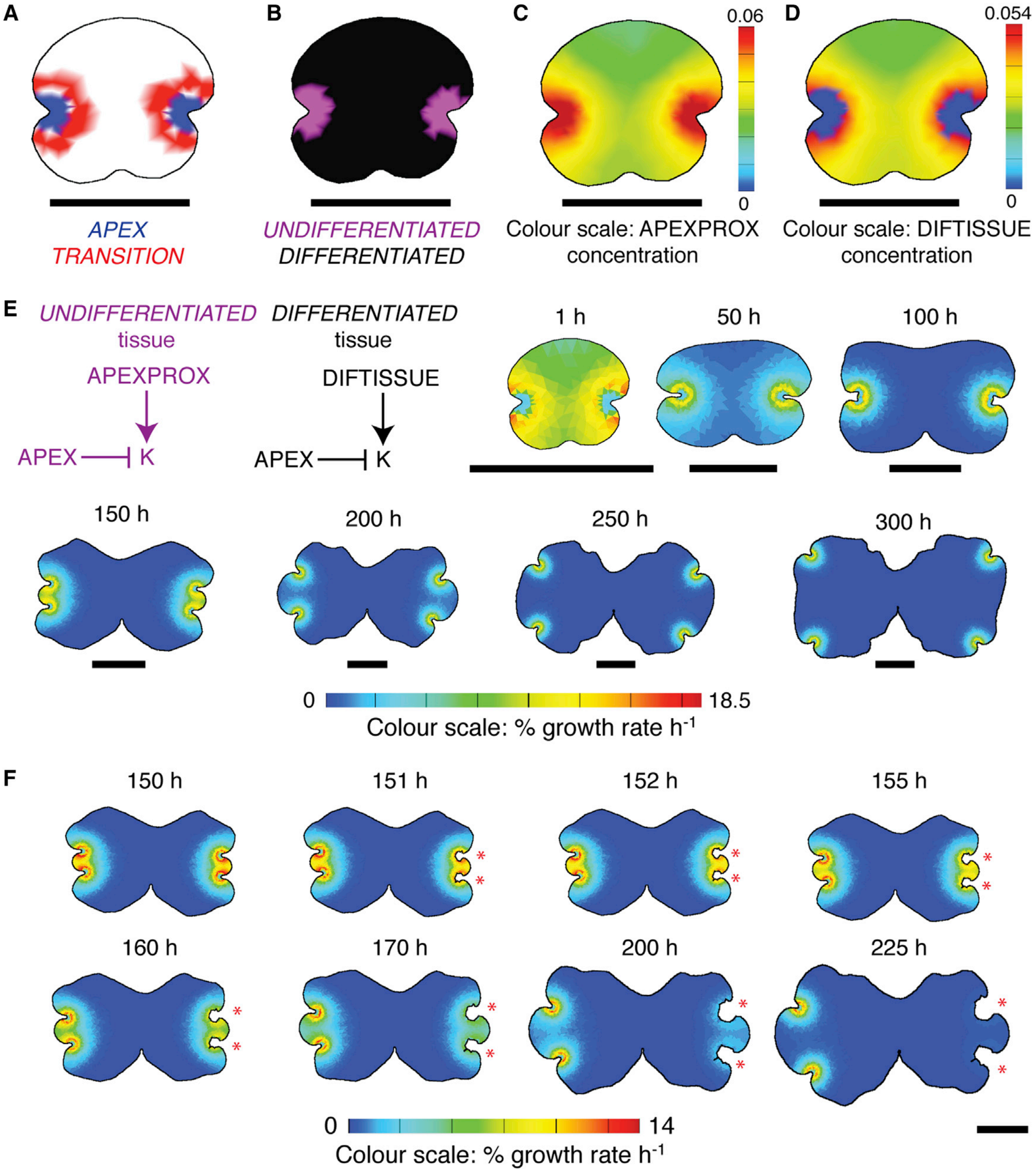

Figure 5. A Notch-Pre-patterns-Growth Model Can Account for the Major Shape Transitions Occurring during Plastochrons 1 and 2 and Thallus Shape Changes following Surgery

(A–D) The distributions of APEX (blue) and TRANSITION (red; A), UNDIFFERENTIATED (purple) and DIFFERENTIATED (black) tissues (B), and APEXPROX (C) and DIFTISSUE (D) on a gemma-shaped starting canvas. Color scales in (C) and (D) indicate morphogen concentration in a.u. Scale bars, 0.5 mm.

(E) Model regulatory network for the notch-pre-patterns-growth model showing growth rules for UNDIFFERENTIATED and DIFFERENTIATED tissues, indicated in purple and black, respectively, and resultant thallus shapes at different time points.

(F) Predicted effects on thallus shape of notch excision (red asterisks) 150 hr into a model simulation. Excision did not cause growth to rapidly cease, rather the cut site edges gradually diverged and the central lobe grew out as in real plants. The color scales represent percentage growth rate per hour. Scale bar, 1 mm.
See also Table S3.

Auxin transport away from the notch has been demonstrated using radiolabelled auxin transport assays and is 2,3,5-triiodobenzoic acid (TIBA) sensitive [30], suggesting that it may be affected by PIN auxin efflux carriers. PIN-mediated auxin transport drives apical cell proliferation and expansive growth in the moss *Physcomitrella patens* [31], and this may be a shared feature of bryophyte gametophytes. However, the nature of PIN dynamics and polarity are not yet clear in non-flowering plants, and callose-based diffusive transport is proposed to have developmental relevance [31–33]. A future challenge will be to dissect specific

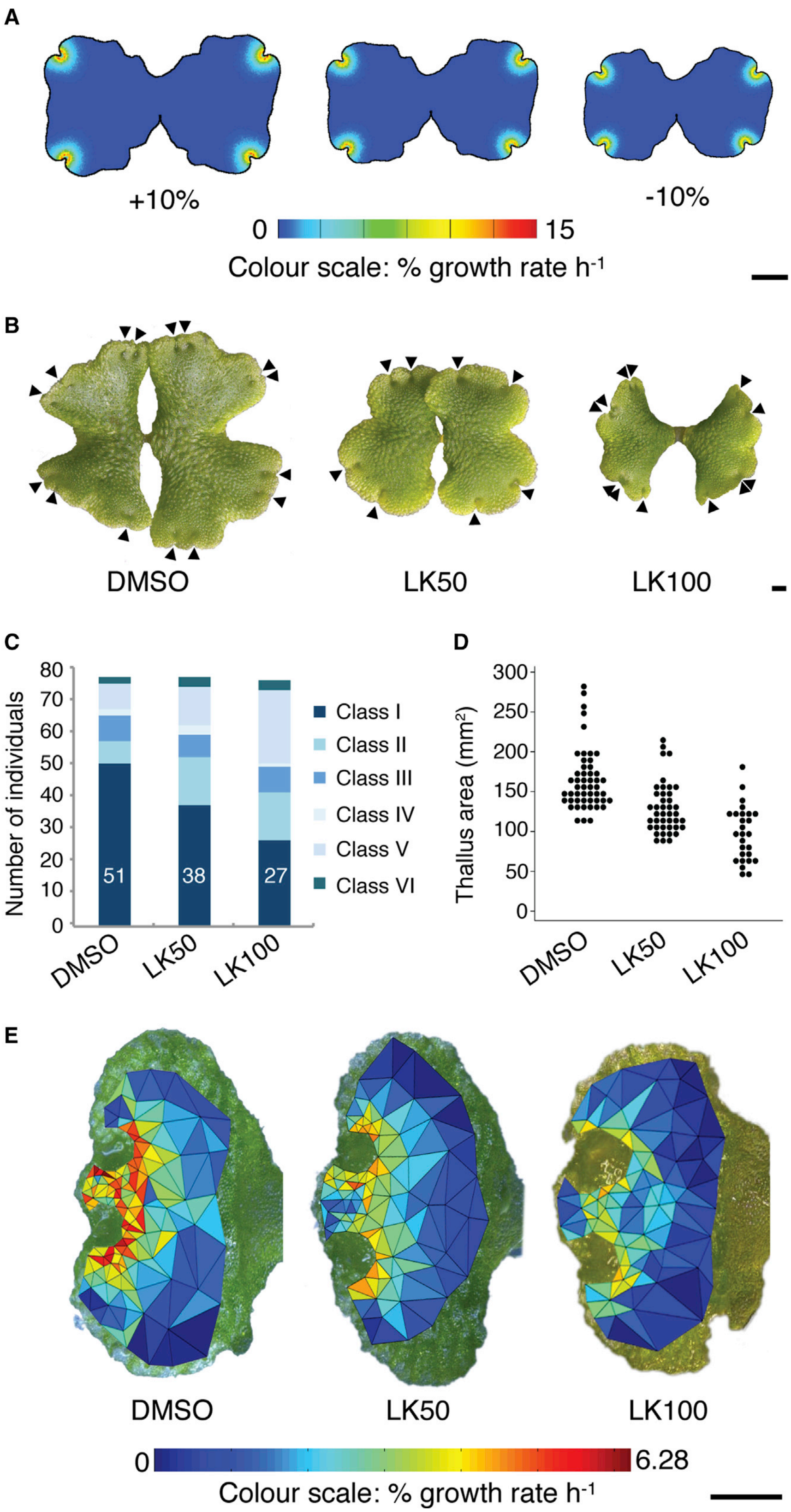

Figure 6. Pharmacological Inhibition of Auxin Biosynthesis Diminishes Thallus Size by Reducing Growth Rates

(A) The effect on model canvas size and growth rate of varying parameter $b_{apexprox}$ (the concentration of APEXPROX at APEX). Scale bar, 1 mm.

(B) Median-sized day 11 thalli grown on medium containing DMSO or L-kynurenine (L-kyn). Arrowheads indicate apical notches. Scale bar, 1 mm.

(C) Column chart showing that the number of thalli with normal development diminishes with increasing L-kyn concentrations. Pooled data from three experimental replicates are shown. The number of class I (normal) thalli for each treatment is shown in white text. Data from individual replicates are given in Figure S5B.

(D) Bee swarm plot of pooled data from three experimental replicates showing the area of thalli from different treatments. ANOVA (after log transformation to better satisfy parametric assumptions) showed that mean growth rate differs between treatments ($F(2,117) = 41.51$; $p << 0.0001$); post hoc Tukey tests showed that all three treatments had significantly different mean growth rates (all pairwise p values were <0.0001). Data from each replicate are given in Figure S5C.

(E) The growth rate distribution in similar-sized control and L-kyn-treated thalli was measured as described for Figure 2. Scale bar, 1 mm.

See also Figure S5.

effects of auxin and its transport and metabolism on thallus growth.

Although planar growth forms evolved before plants' transition to land, multi-layered planar tissues were a land plant innovation [13, 34]. So far analyses of planar tissue growth have focused on flowering plant organs, such as leaves and petals, which grow determinately. A key role for oriented growth responses to convergent or divergent polarity fields has been demonstrated and can generate organ shape diversity [7, 11]. Neither our notch-drives-growth nor our notch-pre-patterns-growth model required such oriented growth. Instead, growth rate heterogeneity with specified isotropic growth was sufficient to account for thallus shape. As identified above, the components contributing to heterogeneity were local growth suppression at the notch (APEX) and differential growth responses in UNDIFFERENTIATED and DIFFERENTIATED tissue regions. Mechanistic understanding of *Marchantia polymorpha* development is in its infancy, but the notch contains the apical stem cells that generate thallus tissues, and we note that slow growth is a feature of meristems in many land plant groups [4]. For instance, the central stem cell zone of flowering plant meristems is stiffer than the more rapidly proliferative zone, and stiffening inhibits growth [35]. We speculate that slow growth at APEX coupled with rapid growth in the APEXPROX-driven UNDIFFERENTIATED zone provides analogous functional zonation.

EXPERIMENTAL PROCEDURES

Plant Material

Marchantia polymorpha male line Takaragaike-1 [19] was grown on half-strength Gamborg's B5 medium [36] containing 0.8% agar. For growth analysis, gemmae were plated on 9 cm Petri dishes and grown in a controlled environment room at 23°C in long-day conditions (8 hr dark and 16 hr light) under white light.

Microscopy, Image, and Growth Analyses

Microscopy and Image Collection

Images of whole plants were taken at time intervals of roughly 24 hr using a Keyence VHX-1000 digital microscope at magnifications of between 20× and 200×. If a plant was larger than the field of view, multiple images were collated in Photoshop.

Image Analyses

The number of apical notches in each image was counted by eye and total thallus area was measured in ImageJ (http://imagej.nih.gov/ij/). The best-fit curves for the thallus area data were calculated using the non-linear fitting function in MATLAB. The growth tensor field across the thallus was calculated as previously described by using Point Tracker software [11], except dorsal air pores rather than cell vertices were used as material points to track. Points were linked to form polygonal regions covering the majority of the mature dorsal thallus tissue. Growth for each region was calculated from its deformation between pairs of consecutive images (Figure S1).

Statistical Growth Analyses

For calculation of the distances between each tissue region and the two apical notches, the coordinates for the centroid of each region were calculated from Point Tracker data. The coordinates of the two apical notches were defined manually. The distance between each centroid and each apical notch was calculated using a custom script in MATLAB. Statistical model fitting was carried out in R [37], using the built-in function nls to fit fixed-effect models (the data were pooled over replicates) and nlme [38] to fit mixed-effect models (model coefficients vary by thallus). The scripts are provided in the "fitAllModels.R" file in Data S1. Correct convergence was tested by checking that fitted values did not depend on the starting values of model coefficients using the "testStartingPoints.R" script provided in Data S1. The adjusted R^2 and AIC were used to assess relative goodness of fit between models (see the Supplemental Experimental Procedures), and model fit was assessed by examining residual plots (Figures S3B and S3C). For visualization of the predictions of the best-fitting model, the predicted growth rates were plotted onto the Point Tracker mesh using custom-written MATLAB scripts.

Modeling Framework

The 2D liverwort thallus models were specified using the GPT framework in *GFtbox* as described in the Supplemental Experimental Procedures and elsewhere [8].

SUPPLEMENTAL INFORMATION

Supplemental Information includes Supplemental Experimental Procedures, five figures, three tables, and one data file and can be found with this article online at http://dx.doi.org/10.1016/j.cub.2016.10.056.

AUTHOR CONTRIBUTIONS

All authors made significant contributions to this work. The project was designed by J.E.S. and C.J.H. J.E.S. did the modeling with help from colleagues acknowledged below. J.E.S. did the experimental work for Figures 1, 2, 3, 4, and 5 and C.J.H. did experiments for Figure 6. Statistical analyses of growth rate distribution data were designed and undertaken by J.E.S. and N.J.C. C.J.H. and J.E.S. wrote the manuscript with help from N.J.C.

ACKNOWLEDGMENTS

We thank Yuki Yasumura and Liz Alvey for providing *Marchantia*. We thank Enrico Coen and Andrew Bangham for help with modeling; Erica Kuchen, Sam Fox, and Pierre Barbier de Reuille for help with Point Tracker; Katie Abley and Richard Kennaway for help with *GFtbox*; and Jacob Newman for computing support. Model simulations were performed on the CamGrid cluster, and we thank Mark Calleja for administering this system and for help accessing it. We thank Stephanie Smith-Unna for help with coding in MATLAB. We thank the BBSRC (BB/F016581/1) for funding J.E.S.'s PhD research and the Gatsby Charitable Foundation (GAT2962) and the Royal Society (RG54416) for funding C.J.H.'s research.

Received: October 14, 2015
Revised: October 4, 2016
Accepted: October 27, 2016
Published: December 8, 2016

REFERENCES

1. Niklas, K.J., and Kerchner, V. (1984). Mechanical and photosynthetic constraints on the evolution of plant shape. Paleobiology *10*, 79–101.
2. Floyd, S.K., and Bowman, J.L. (2007). The ancestral developmental tool kit of land plants. Int. J. Plant Sci. *168*, 1–35.
3. Kalisz, S., and Kramer, E.M. (2008). Variation and constraint in plant evolution and development. Heredity (Edinb) *100*, 171–177.
4. Steeves, T.A., and Sussex, I.M. (1989). Patterns in Plant Development (Cambridge University Press).
5. Parihar, N.S. (1967). Bryophyta (Indian Universities Press).
6. Coen, E., Rolland-Lagan, A.-G., Matthews, M., Bangham, J.A., and Prusinkiewicz, P. (2004). The genetics of geometry. Proc. Natl. Acad. Sci. USA *101*, 4728–4735.
7. Sauret-Güeto, S., Schiessl, K., Bangham, A., Sablowski, R., and Coen, E. (2013). JAGGED controls *Arabidopsis* petal growth and shape by interacting with a divergent polarity field. PLoS Biol. *11*, e1001550.
8. Kennaway, R., Coen, E., Green, A., and Bangham, A. (2011). Generation of diverse biological forms through combinatorial interactions between tissue polarity and growth. PLoS Comput. Biol. *7*, e1002071.
9. Rolland-Lagan, A.G., Bangham, J.A., and Coen, E. (2003). Growth dynamics underlying petal shape and asymmetry. Nature *422*, 161–163.

10. Green, A.A., Kennaway, J.R., Hanna, A.I., Bangham, J.A., and Coen, E. (2010). Genetic control of organ shape and tissue polarity. PLoS Biol. *8*, e1000537.
11. Kuchen, E.E., Fox, S., de Reuille, P.B., Kennaway, R., Bensmihen, S., Avondo, J., Calder, G.M., Southam, P., Robinson, S., Bangham, A., and Coen, E. (2012). Generation of leaf shape through early patterns of growth and tissue polarity. Science *335*, 1092–1096.
12. Aigouy, B., Farhadifar, R., Staple, D.B., Sagner, A., Röper, J.C., Jülicher, F., and Eaton, S. (2010). Cell flow reorients the axis of planar polarity in the wing epithelium of *Drosophila*. Cell *142*, 773–786.
13. Graham, L.E., Cook, M.E., and Busse, J.S. (2000). The origin of plants: body plan changes contributing to a major evolutionary radiation. Proc. Natl. Acad. Sci. USA *97*, 4535–4540.
14. Langdale, J.A., and Harrison, C.J. (2008). Developmental changes during the evolution of plant form. In Evolving Pathways: Key Themes in Evolutionary Developmental Biology, A.M.G. Fusco, ed. (Cambridge University Press), pp. 299–315.
15. Wellman, C.H., Osterloff, P.L., and Mohiuddin, U. (2003). Fragments of the earliest land plants. Nature *425*, 282–285.
16. Gerrienne, P., Dilcher, D.L., Bergamaschi, S., Milagres, I., Pereira, E., and Rodrigues, M.A.C. (2006). An exceptional specimen of the early land plant *Cooksonia paranensis*, and a hypothesis on the life cycle of the earliest eutracheophytes. Rev. Palaeobot. Palynol. *142*, 123–130.
17. Qiu, Y.-L., Li, L., Wang, B., Chen, Z., Knoop, V., Groth-Malonek, M., Dombrovska, O., Lee, J., Kent, L., Rest, J., et al. (2006). The deepest divergences in land plants inferred from phylogenomic evidence. Proc. Natl. Acad. Sci. USA *103*, 15511–15516.
18. Hernick, L.V., Landing, E., and Bartowski, K.E. (2008). Earth's oldest liverworts—*Metzgeriothallus sharonae* sp. nov. from the middle Devonian (Givetian) of eastern New York, USA. Rev. Palaeobot. Palynol. *148*, 154–162.
19. Ishizaki, K., Chiyoda, S., Yamato, K.T., and Kohchi, T. (2008). Agrobacterium-mediated transformation of the haploid liverwort Marchantia polymorpha L., an emerging model for plant biology. Plant Cell Physiol. *49*, 1084–1091.
20. Ishizaki, K., Nishihama, R., Yamato, K.T., and Kohchi, T. (2016). Molecular genetic tools and techniques for *Marchantia polymorpha* research. Plant Cell Physiol. *57*, 262–270.
21. Tarén, N. (1958). Factors regulating the initial development of gemmae in Marchantia polymorpha. Bryologist *61*, 191–204.
22. Akaike, H. (1974). A new look at the statistical model identification. IEEE Trans. Automat. Contr. *19*, 716–723.
23. Burnham, K.P., and Anderson, D.R. (2004). Multimodel inference understanding AIC and BIC in model selection. Sociol. Methods Res. *33*, 261–304.
24. Flores-Sandoval, E., Eklund, D.M., and Bowman, J.L. (2015). A simple auxin transcriptional response system regulates multiple morphogenetic processes in the liverwort *Marchantia polymorpha*. PLoS Genet. *11*, e1005207.
25. Eklund, D.M., Ishizaki, K., Flores-Sandoval, E., Kikuchi, S., Takebayashi, Y., Tsukamoto, S., Hirakawa, Y., Nonomura, M., Kato, H., Kouno, M., et al. (2015). Auxin produced by the indole-3-pyruvic acid pathway regulates development and gemmae dormancy in the liverwort *Marchantia polymorpha*. Plant Cell *27*, 1650–1669.
26. Bhalerao, R.P., and Bennett, M.J. (2003). The case for morphogens in plants. Nat. Cell Biol. *5*, 939–943.
27. Benková, E., Ivanchenko, M.G., Friml, J., Shishkova, S., and Dubrovsky, J.G. (2009). A morphogenetic trigger: is there an emerging concept in plant developmental biology? Trends Plant Sci. *14*, 189–193.
28. Allsopp, A., Pearman, C., and Rao, A. (1968). The effects of some growth substances and inhibitors on the development of Marchantia gemmae. Phytomorphology *18*, 84–94.
29. Ishizaki, K., Nonomura, M., Kato, H., Yamato, K.T., and Kohchi, T. (2012). Visualization of auxin-mediated transcriptional activation using a common auxin-responsive reporter system in the liverwort Marchantia polymorpha. J. Plant Res. *125*, 643–651.
30. Gaal, D.J., Dufresne, S.J., and Maravolo, N.C. (1982). Transport of ^{14}C-indoleacetic acid in the hepatic Marchantia polymorpha. Bryologist *85*, 410–418.
31. Bennett, T.A., Liu, M.M., Aoyama, T., Bierfreund, N.M., Braun, M., Coudert, Y., Dennis, R.J., O'Connor, D., Wang, X.Y., White, C.D., et al. (2014). Plasma membrane-targeted PIN proteins drive shoot development in a moss. Curr. Biol. *24*, 2776–2785.
32. Viaene, T., Landberg, K., Thelander, M., Medvecka, E., Pederson, E., Feraru, E., Cooper, E.D., Karimi, M., Delwiche, C.F., Ljung, K., et al. (2014). Directional auxin transport mechanisms in early diverging land plants. Curr. Biol. *24*, 2786–2791.
33. Coudert, Y., Palubicki, W., Ljung, K., Novak, O., Leyser, O., and Harrison, C.J. (2015). Three ancient hormonal cues co-ordinate shoot branching in a moss. eLife *4*, e06808.
34. Bremer, K., Humphries, C.J., Mishler, B.D., and Churchill, S.P. (1987). On cladistic relationships in green plants. Taxon *36*, 339–349.
35. Kierzkowski, D., Nakayama, N., Routier-Kierzkowska, A.L., Weber, A., Bayer, E., Schorderet, M., Reinhardt, D., Kuhlemeier, C., and Smith, R.S. (2012). Elastic domains regulate growth and organogenesis in the plant shoot apical meristem. Science *335*, 1096–1099.
36. Gamborg, O.L., Miller, R.A., and Ojima, K. (1968). Nutrient requirements of suspension cultures of soybean root cells. Exp. Cell Res. *50*, 151–158.
37. R Development Core Team (2015). R: a language and environment for statistical computing (R Foundation for Statistical Computing).
38. Pinheiro, J., Bates, D., DebRoy, S., and Sarkar, D.; R Development Core Team (2015). nlme: linear and nonlinear mixed effects models. R package version 3.1-120. (R Foundation for Statistical Computing).

Current Biology, Volume *27*

Supplemental Information

Regional Growth Rate Differences Specified by Apical Notch Activities Regulate Liverwort Thallus Shape

Jeremy E. Solly, Nik J. Cunniffe, and C. Jill Harrison

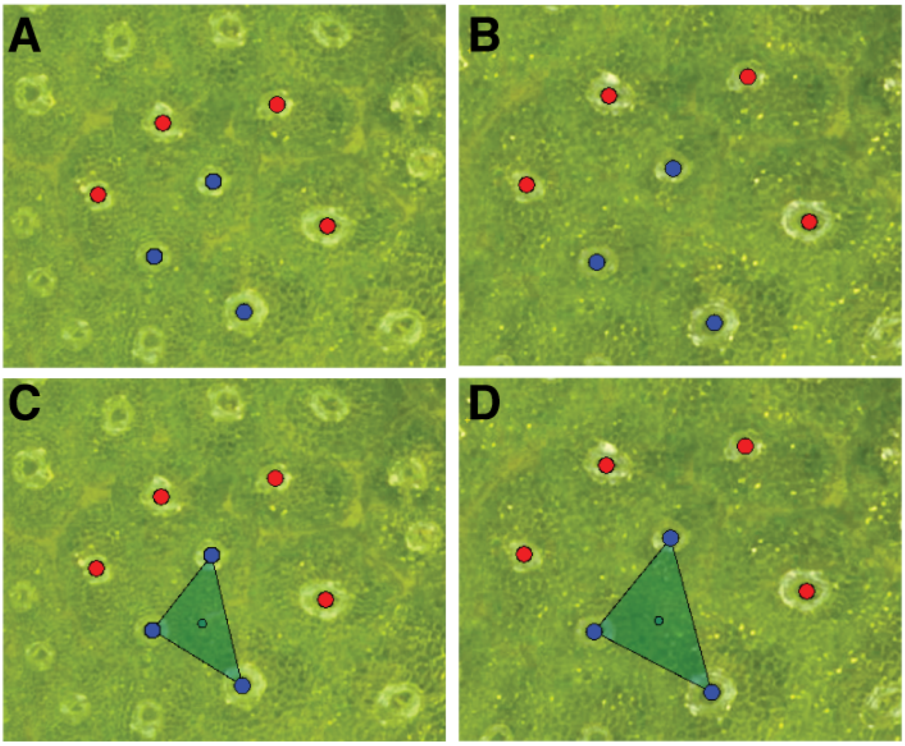

Figure S1 related to Figure 2: Quantification of thallus growth rates using Point Tracker software. (A,B) Points were manually placed on air pores in the first (A) and subsequent (B) images of a time series. (C, D) The points in blue demarcate a triangular region of thallus tissue, and the displacement of points over time (D) was used to calculate areal growth rates.

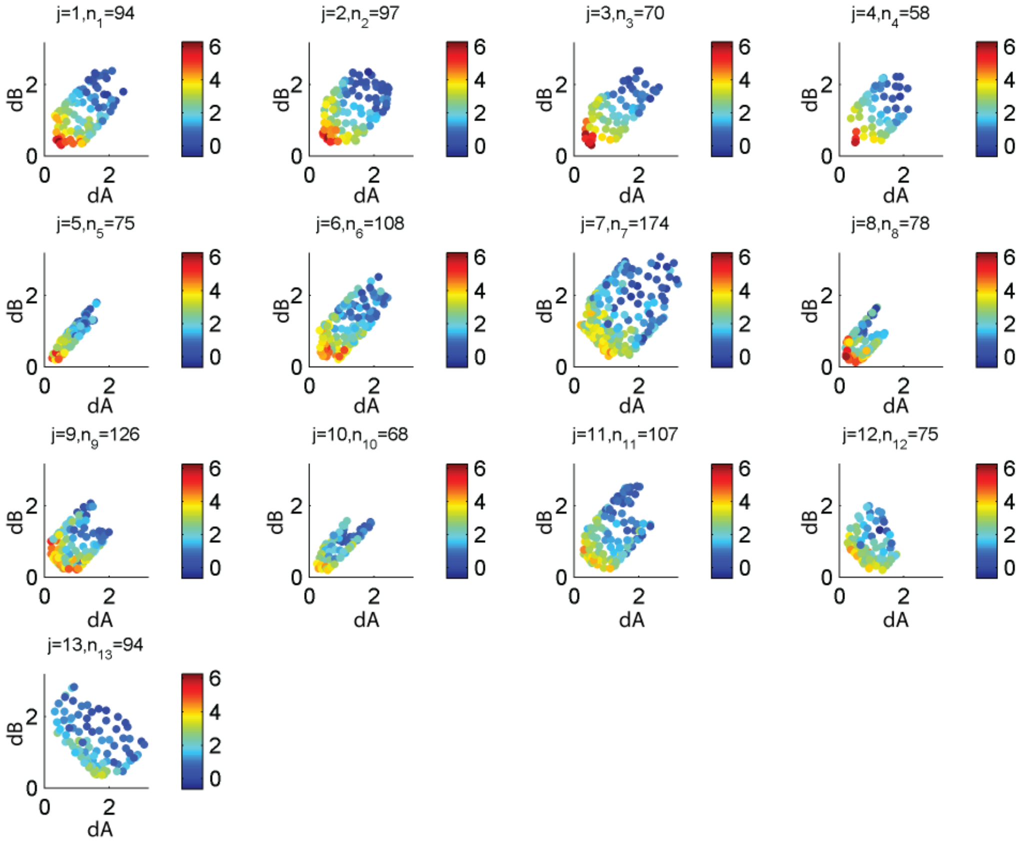

Figure S2 related to Figure 2: Growth rate distributions relative to apical notches generated from 13 half thalli. Each graph represents the growth rate data for one half-thallus over 24 h and dA and dB denote the distances from each notch in mm. j denotes dataset number and n_j denotes the number of points in each dataset. Percentage growth rates are represented by a colour scale with units per hour.

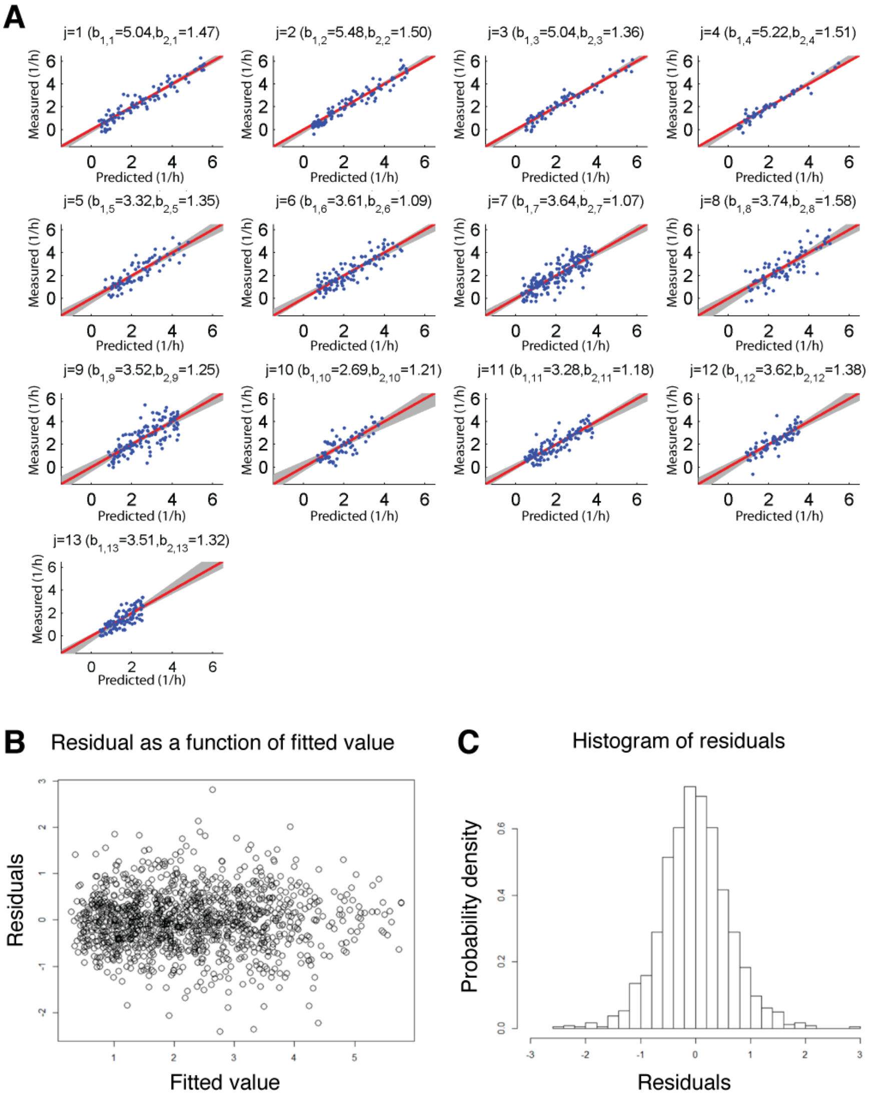

Figure S3 related to Figure 2: Fit of Model 2 (positional hypothesis, exponential decay) to all 13 datasets, showing the relationship between predicted and measured percentage growth rates and model diagnostics. (A) j denotes the dataset number. $b_{1,j}$ and $b_{2,j}$ denote the values of the parameters b_1 and b_2 used to fit the model to each dataset, j. The red lines show $y = x$, the values at which the measured and predicted growth rates exactly match. The grey shading shows the 95% confidence interval of the best-fit line for each dataset. As the red line always falls inside the grey region, the model predicted growth rates well across all 13 datasets. The general equation to calculate predicted growth rates was $k = b_1\left[\exp\left(-b_2 d_A\right) + \exp\left(-b_2 d_B\right)\right]$, where k denotes percentage growth rate, b_1 and b_2 denote parameters estimated to give the best fit for each dataset and d_A and d_B denote distance from notch A and notch B, respectively. (B) Residual-fitted plot, showing the size of residuals does not depend on the fitted value. (C) Empirical probability distribution for model residuals, showing the assumption of a normal distribution was reasonable, despite slight leptokurtic behaviour (a fat tail).

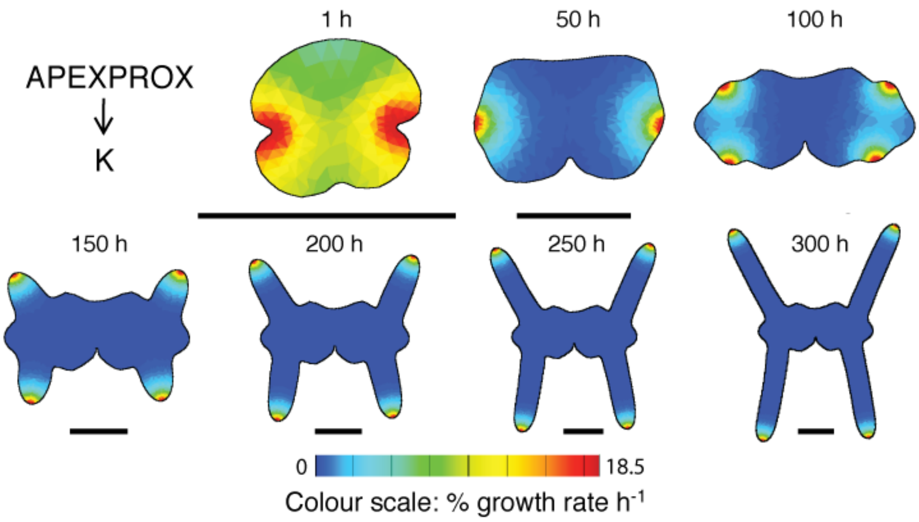

Figure S4 related to Figure 3: Growth suppression at APEX is required to maintain an invaginated notch in the notch-drives-growth model. A diffusible morphogen, APEXPROX was produced at APEX and distributed across the canvas. The model regulatory network is shown in the top left corner. The colour scale denotes percentage growth rate per hour. Scale bar =1 mm.

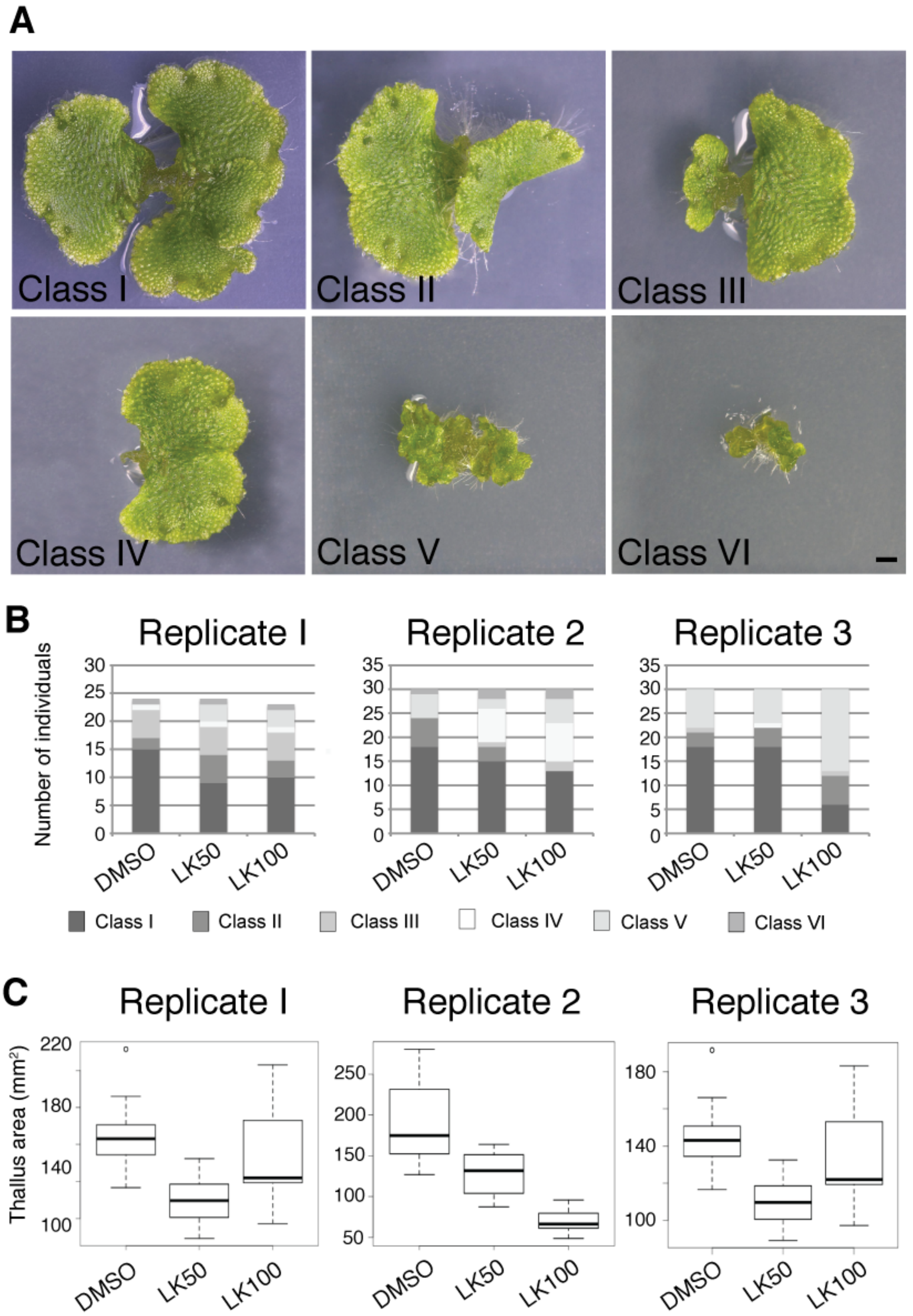

Figure S5 related to Figure 6: Developmental perturbations in thalli grown on L-kyn. (A) Class I thalli were normal, Class II thalli had polarity reversals, Class II thalli had a strong growth mismatch between thallus halves, Class IV plants only grew half a thallus, Class V thalli had differentiation defects and Class VI thalli didn't grow. Class I thalli from each treatment were included in growth analyses. Scale bar = 0.5 mm (B) The proportion of thalli with Class II to Class VI developmental defects varied between pharmacological treatments in three experimental replicates. (C) Class I thallus area varied by treatment in each experimental replicate.

	Model	Fitted parameters	ΔAIC	R^2
Model 1 (Mixed)	$k_{ij}=b_{1j}-b_{2j}\left(d_{A,ij}+d_{B,ij}\right)+\varepsilon_{ij}$<br>$\begin{pmatrix} b_{1j}\\ b_{2j}\end{pmatrix}\sim N\left(\begin{pmatrix} b_1\\ b_2\end{pmatrix},\begin{pmatrix}\sigma_1^2 & \rho\sigma_1\sigma_2\\ \rho\sigma_1\sigma_2 & \sigma_2^2\end{pmatrix}\right)$<br>$\varepsilon_{ij}\sim N(0,\sigma^2)$	$b_1=5.00\,(4.59-5.42)$<br>$b_2=1.26\,(1.10-1.43)$<br>$\sigma_1=0.73\,(0.48-1.11)$<br>$\sigma_2=0.29\,(0.19-0.44)$<br>$\rho=0.79\,(0.45-0.93)$	266.0	0.73
Model 1 (Pooled)	$k_i=b_1-b_2\left(d_{A,i}+d_{B,i}\right)+\varepsilon_i$<br>$\varepsilon_i\sim N(0,\sigma^2)$	$b_1=4.58\,(4.47-4.70)$<br>$b_2=1.03\,(0.98-1.07)$	609.3	0.61
Model 2 (Mixed)	$k_{ij}=b_{1j}\left(\exp(-b_{2j}d_{A,ij})+\exp(-b_{2j}d_{B,ij})\right)+\varepsilon_{ij}$<br>$\begin{pmatrix} b_{1j}\\ b_{2j}\end{pmatrix}\sim N\left(\begin{pmatrix} b_1\\ b_2\end{pmatrix},\begin{pmatrix}\sigma_1^2 & \rho\sigma_1\sigma_2\\ \rho\sigma_1\sigma_2 & \sigma_2^2\end{pmatrix}\right)$<br>$\varepsilon_{ij}\sim N(0,\sigma^2)$	$b_1=3.98\,(3.46-4.49)$<br>$b_2=1.33\,(1.22-1.44)$<br>$\sigma_1=0.90\,(0.59-1.36)$<br>$\sigma_2=0.18\,(0.11-0.29)$<br>$\rho=0.64\,(0.11-0.88)$	0	0.78
Model 2 (Pooled)	$k_i=b_1\left(\exp(-b_2d_{A,i})+\exp(-b_2d_{B,i})\right)+\varepsilon_i$<br>$\varepsilon_i\sim N(0,\sigma^2)$	$b_1=3.54\,(3.39-3.69)$<br>$b_2=1.20\,(1.14-1.26)$	355.4	0.68
Model 3 (Mixed)	$k_{ij}=b_{1j}-b_{2j}d_{M,ij}+\varepsilon_{ij}$<br>$\begin{pmatrix} b_{1j}\\ b_{2j}\end{pmatrix}\sim N\left(\begin{pmatrix} b_1\\ b_2\end{pmatrix},\begin{pmatrix}\sigma_1^2 & \rho\sigma_1\sigma_2\\ \rho\sigma_1\sigma_2 & \sigma_2^2\end{pmatrix}\right)$<br>$\varepsilon_{ij}\sim N(0,\sigma^2)$	$b_1=4.34\,(3.95-4.74)$<br>$b_2=2.53\,(2.15-2.90)$<br>$\sigma_1=0.70\,(0.46-1.05)$<br>$\sigma_2=0.66\,(0.43-1.01)$<br>$\rho=0.81\,(0.49-0.93)$	433.8	0.69
Model 3 (Pooled)	$k_i=b_1-b_2d_{M,i}+\varepsilon_i$<br>$\varepsilon_i\sim N(0,\sigma^2)$	$b_1=4.06\,(3.96-4.16)$<br>$b_2=2.11\,(2.01-2.21)$	716.0	0.58
Model 4 (Mixed)	$k_{ij}=b_{1j}\exp(-b_{2j}d_{M,ij})+\varepsilon_{ij}$<br>$\begin{pmatrix} b_{1j}\\ b_{2j}\end{pmatrix}\sim N\left(\begin{pmatrix} b_1\\ b_2\end{pmatrix},\begin{pmatrix}\sigma_1^2 & \rho\sigma_1\sigma_2\\ \rho\sigma_1\sigma_2 & \sigma_2^2\end{pmatrix}\right)$<br>$\varepsilon_{ij}\sim N(0,\sigma^2)$	$b_1=6.28\,(5.35-7.21)$<br>$b_2=1.35\,(1.21-1.50)$<br>$\sigma_1=1.65\,(1.08-2.52)$<br>$\sigma_2=0.24\,(0.15-0.38)$<br>$\rho=0.78\,(0.38-0.93)$	196.0	0.74
Model 4 (Pooled)	$k_i=b_1\exp(-b_2d_{M,i})+\varepsilon_i$<br>$\varepsilon_i\sim N(0,\sigma^2)$	$b_1=5.76\,(5.53-5.99)$<br>$b_2=1.26\,(1.20-1.33)$	492.9	0.65

Table S1 related to Figure 2. Comparison of models fitted in growth analyses. In the mixed models, k_{ij} is the fitted growth rate for the ith observation in the jth group (i.e. the ith location on the jth thallus), $d_{A,ij}$ is the distance from notch A for that observation, $d_{B,ij}$ is the distance from notch B, $d_{M,ij}=\min(d_{A,ij},d_{B,ij})$, ε_{ij} is the error, and the parameters b_{1j} and b_{2j} vary with j via a distributional

assumption. In the pooled models the fitted growth rate for the ith observation (pooled over all datasets) is k_i, $d_{A,i}$ is the distance from notch A for that observation, $d_{B,i}$ is the distance from notch B, $d_{M,i} = \min(d_{A,i}, d_{B,i})$, the error is ε_i, and the parameters to be estimated are simply b_1 and b_2. Note that k_{ij} and k_i always have units 1/h, b_1 and b_{1j} always have units 1/h, and σ_1^2 always has units $(1/h)^2$. The units of other parameters depend on the model in question. In both variants of models 1 and 3, b_2 and b_{2j} have units 1/(h mm), and σ_2^2 has units $(1/(h\ mm))^2$. However, in both variants of models 2 and 4, b_2 and b_{2j} have units 1/mm, and σ_2^2 has units $(1/mm)^2$. All fitted values are reported with 95% confidence intervals in brackets. The value of R^2 reported for each fitted model was calculated as the squared correlation between the predicted and fitted values. ΔAIC is reported relative to the Model Two (mixed), the best-fitting model.

Parameter	Description	Value	Effect of changing value
$b_{apexprox}$	APEXPROX concentration at APEX	0.06	Same shape transitions, different timing, different canvas size (larger values give bigger canvas).
$D_{apexprox}$	APEXPROX diffusion rate	0.03 $mm^2\ h^{-1}$	Same shape transtitions, different canvas size (larger values give bigger canvas).
$\mu_{apexprox}$	APEXPROX decay rate	0.8 h^{-1}	Same shape transitions, different canvas size (larger values give smaller canvas).
h_{apex}	Inhibition coefficient for APEX	5	The extent of notch invagination. The minimum value required to make a notch was used.
$t_{branching}$	Branching time	80 h	Affects the timing of shape transitions, with a minor effect on canvas shape.

Table S2 related to Figure 3: List of parameters used in the notch-drives-growth model, and effect of changing parameter values on thallus shape.

Parameter	Description	Value	Effect of changing value
$b_{apexprox}$	APEXPROX concentration at APEX	0.06	Same shape transitions, different timing, different canvas size (larger values give bigger canvas).
$D_{apexprox}$	APEXPROX diffusion rate	0.03 $mm^2\ h^{-1}$	Same shape transtitions, different canvas size (larger values give bigger canvas).
$\mu_{apexprox}$	APEXPROX decay rate	0.8 h^{-1}	Same shape transitions, different canvas size (larger values give smaller canvas).
$\mu_{diftissue}$	DIFTISSUE decay rate	0.04 h^{-1}	Same shape transitions, different canvas size (larger values give smaller canvas).
h_{apex}	Inhibition coefficient for APEX	5	The extent of notch invagination. The minimum value required to make a notch was used.
$t_{branching}$	Branching time	80 h	Affects the timing of shape transitions, with a minor effect on canvas shape.

Table S3 related to Figure 5: List of parameters used in the notch-pre-patterns-growth model, and effect of changing parameter values on thallus shape.

Supplemental Experimental Procedures

Thallus shape modelling

Model overview

Liverwort thallus growth was modelled using the Growing Polarised Tissue (GPT) framework in *GFtbox*, a MATLAB application [S1]. Model runs involved simulating tissue growth by calculating the deformation of a canvas, which represented liverwort thallus tissue. The canvas was made up of a finite element mesh and the pattern of deformation depended on the distributions of growth-modulating factors over the canvas.

For both models described here, the initial canvas was formed in the shape of a gemma, consisted of around 440 elements and was roughly 0.5 mm wide. Simulations ran for up to 300 virtual hours and outputs were generated during growth. Finite elements were subdivided during growth.

Factors

Notional growth regulators were represented in the models as factors, which were distributed over the canvas as described elsewhere [S1]. Factors were either diffusible (able to propagate through the canvas) or fixed (immobile on the canvas). In equations, diffusible factors are denoted by a bold letter **s** subscripted with the factor name, while fixed factors are denoted by **i** subscripted with the factor name. For example, the diffusible factor APEXPROX is denoted by $\mathbf{s}_{apexprox}$ in equations, whereas the fixed factor APEX is denoted by $\mathbf{i}_{apex}$.

Initial distributions of factors were established during a setup phase from -9 h to 0 h. Factors had a single value for each vertex on the canvas and values between vertices were linearly interpolated across each finite element.

Parameters

The parameters and parameter values used in model simulations are shown in Tables S2 and S3. A range of parameter values for each parameter was explored, and values included here were selected by their capacity to reproduce shape transitions identified in Figure 1. The effect of varying parameter values was explored and is described in Tables S2 and S3.

Models

The pattern of deformation in each model was affected by the distributions of factors. Factors could interact with each other and influence specified growth rates. The model Gene Regulatory Network (GRN) determined the interactions between factors. The Growth rate Regulatory Network (KRN) determined how factors influenced specified growth rates.

GRN: 'notch-drives-growth' model

Two discrete regions of the fixed factor APEX were expressed at a level of one at the base of each apical notch on the initial canvas and zero elsewhere. The diffusible factor APEXPROX was fixed at a value of 0.06 ($b_{apexprox}$) where APEX was expressed and diffused according to the equation:

$$\frac{\partial \mathbf{s}_{apexprox}}{\partial t} = D_{apexprox} \nabla^2 \mathbf{s}_{apexprox} - \mu_{apexprox} \mathbf{s}_{apexprox} ,$$

where $\mathbf{s}_{apexprox}$ is the concentration of APEXPROX, $D_{apexprox}$ is its diffusion rate and $\mu_{apexprox}$ its decay rate across the canvas. The APEXPROX distribution was allowed to establish during the setup phase for 9 h of virtual time before growth commenced and APEXPROX propagation continued throughout growth.

At time 80 h ($t_{branching}$), branching was imposed on the apices. The single region of APEX at the base of each notch was replaced by two discrete regions, which expressed APEX at a level of one. APEXPROX continued to be fixed at a concentration of $b_{apexprox}$ where APEX was expressed.

GRN: 'notch-pre-patterns-growth' model

The distributions of APEX and APEXPROX were identical in both models. Four new factors were included in the 'notch-pre-patterns-growth' model. TRANSITION was positioned in a ring around each region of APEX. It was expressed at a level of one in any vertex that was between 0.08 and 0.14 mm from the centre of APEX. TRANSITION defined whether canvas regions expressed the factors DIFFERENTIATED or UNDIFFERENTIATED. Regions that were closer to APEX than TRANSITION or included APEX expressed UNDIFFERENTIATED at a level of one and DIFFERENTIATED was zero. Regions that expressed TRANSITION or were further from APEX than TRANSITION expressed DIFFERENTIATED at a level of one and UNDIFFERENTIATED was zero.

TRANSITION also promoted the production of DIFTISSUE. In regions expressing TRANSITION, the concentration of DIFTISSUE was set as equal to the value of APEXPROX. DIFTISSUE subsequently decayed according to the equation:

$$\frac{\partial \mathbf{s}_{diftissue}}{\partial t} = -\mu_{diftissue} \mathbf{s}_{diftissue} ,$$

where $\mathbf{s}_{diftissue}$ is the concentration of DIFTISSUE and $\mu_{diftissue}$ is its decay rate. The diffusion rate of DIFTISSUE was set as zero.

The initial distribution of DIFTISSUE was set up manually as an apical-basal gradient across DIFFERENTIATED-expressing tissue. Once the canvas deformation commenced at time 0 h,

DIFTISSUE was produced only in tissue expressing TRANSITION and decayed in tissue not expressing TRANSITION.

KRN: 'notch-drives-growth' model

In *GFtbox* growth is calculated using two equations, which specify growth rates parallel (K_{par}) and perpendicular (K_{per}) to a polarity gradient, if present. If no polarity is present, specified growth is isotropic and its value is the average of the outputs of the two growth equations. Polarity was not invoked in these models, so the two equations were identical. Here, K_{par} and K_{per} are generalised as K for simplicity.

In the 'notch-drives-growth' model, K was proportional to the concentration of APEXPROX across the canvas. The function *inh* was used to enable growth inhibition at APEX and took the form:

$$inh\left(h_{apex},\mathbf{i}_{apex}\right)=\frac{1}{1+h_{apex}\mathbf{i}_{apex}},$$

where h_{apex} is the inhibition of growth by APEX and took a value of 5. Thus, the growth equation was:

$$K=\mathbf{s}_{apexprox}\bullet inh\left(h_{apex},\mathbf{i}_{apex}\right).$$

KRN: 'notch-pre-patterns-growth' model

In the 'notch-pre-patterns-growth' model, growth was regulated differently depending on whether tissue expressed DIFFERENTIATED or UNDIFFERENTIATED. In UNDIFFERENTIATED tissue close to APEX, growth rate was proportional to APEXPROX. In DIFFERENTIATED tissue, growth rate was instead proportional to DIFTISSUE:

$$K=\left(\mathbf{s}_{apexprox}\mathbf{i}_{undifferentiated}+\mathbf{s}_{diftissue}\mathbf{i}_{differentiated}\right)\bullet inh\left(h_{apex},\mathbf{i}_{apex}\right).$$

Supplemental Reference

S1. Kennaway, R., Coen, E., Green, A., and Bangham, A. (2011). Generation of diverse biological forms through combinatorial interactions between tissue polarity and growth. PloS Computational Biology *7*, e1002071.
